# Beyond basic characterization and omics: Immunomodulatory roles of platelet‐derived extracellular vesicles unveiled by functional testing

**DOI:** 10.1002/jev2.12513

**Published:** 2024-09-27

**Authors:** Mari Palviainen, Johanna Puutio, Rikke Halse Østergaard, Johannes A. Eble, Katariina Maaninka, Umar Butt, Joseph Ndika, Otto K. Kari, Masood Kamali‐Moghaddam, Kasper Kjaer‐Sorensen, Claus Oxvig, Ana M. Aransay, Juan M. Falcon‐Perez, Antonio Federico, Dario Greco, Saara Laitinen, Yuya Hayashi, Pia R.‐M. Siljander

**Affiliations:** ^1^ EV Group, Molecular and Integrative Biosciences Research Programme, Faculty of Biological and Environmental Sciences, and CURED, Drug Research Program, Faculty of Pharmacy University of Helsinki Helsinki Finland; ^2^ EV Core, Molecular and Integrative Biosciences Research Programme, Faculty of Biological and Environmental Sciences University of Helsinki Helsinki Finland; ^3^ Department of Molecular Biology and Genetics Aarhus University Aarhus Denmark; ^4^ Institute of Physiological Chemistry and Pathobiochemistry University of Münster Münster Germany; ^5^ Drug Research Program, Faculty of Pharmacy University of Helsinki Helsinki Finland; ^6^ Department of Immunology, Genetics and Pathology, Science for Life Laboratory Uppsala University Uppsala Sweden; ^7^ Genome Analysis Platform, Center for Cooperative Research in Biosciences Basque Research and Technology Alliance (BRTA) Mendaro Spain; ^8^ Centro de Investigación Biomédica en Red de Enfermedades Hepáticas Y Digestivas (CIBERehd) Madrid Spain; ^9^ Exosomes Laboratory and Metabolomics Platform, Center for Cooperative Research in Biosciences (CIC bioGUNE) Basque Research and Technology Alliance (BRTA) Derio Spain; ^10^ Ikerbasque, Basque Foundation for Science Bilbao Spain; ^11^ Finnish Hub for Development and Validation of Integrated Approaches (FHAIVE); Faculty of Medicine and Health Technology Tampere University Tampere Finland; ^12^ Division of Pharmaceutical Biosciences, Faculty of Pharmacy University of Helsinki Helsinki Finland; ^13^ Research and Development Finnish Red Cross Blood Service (FRCBS) Helsinki Finland; ^14^ Interdisciplinary Nanoscience Center (iNANO) Aarhus University Aarhus Denmark

**Keywords:** extracellular vesicles, functional assays, immunomodulatory, inflammation, innate immunity, macrophages, miRNA, platelets, protein corona

## Abstract

Renowned for their role in haemostasis and thrombosis, platelets are also increasingly recognized for their contribution in innate immunity, immunothrombosis and inflammatory diseases. Platelets express a wide range of receptors, which allows them to reach a variety of activation endpoints and grants them immunomodulatory functions. Activated platelets release extracellular vesicles (PEVs), whose formation and molecular cargo has been shown to depend on receptor‐mediated activation and environmental cues.

This study compared the immunomodulatory profiles of PEVs generated via activation of platelets by different receptors, glycoprotein VI, C‐type lectin‐like receptor 2 and combining all thrombin‐collagen receptors. Functional assays in vivo in zebrafish and in vitro in human macrophages highlighted distinct homing and secretory responses triggered by the PEVs. In contrast, omics analyses of protein and miRNA cargo combined with physicochemical particle characterization found only subtle differences between the activated PEV types, which were insufficient to predict their different immunomodulatory functions. In contrast, constitutively released PEVs, formed in the absence of an exogenous activator, displayed a distinct immunomodulatory profile from the receptor‐induced PEVs.

Our findings underscore that PEVs are tunable through receptor‐mediated activation. To truly comprehend their role(s) in mediating platelet functions among immune cells, conducting functional assays is imperative.

## Graphical abstract

This study investigated the immunomodulatory profiles of platelet‐derived extracellular vesicles (PEVs) generated through activation by different platelet receptors: glycoprotein VI (GPVI), C‐type lectin‐like receptor 2 (CLEC‐2), and thrombin‐collagen receptors (TC). In vivo (zebrafish) and in vitro (human macrophage) assays revealed distinct homing patterns and secretory responses for each PEV type, respectively. While particle characterization and analysis of protein and miRNA cargoes identified subtle differences between the PEV types, these variations were insufficient to predict their immunomodulatory effects. Notably, PEVs from unstimulated platelets (US PEVs) exhibited a distinct profile compared to receptor‐induced PEVs. Overall, the findings suggest that PEV immunomodulatory functions are finely tuned, emphasizing the need for functional assays to fully elucidate their roles in platelet‐mediated immune responses.

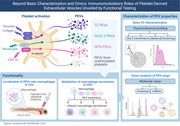

## 1 INTRODUCTION

Platelets, anucleate cells originating from megakaryocytes, exhibit heterogeneity in molecular content and functional profiles. They express receptors on their plasma membrane, which enable versatile signaling‐dependent activation endpoints ranging from adhesion to thrombus formation and procoagulant transformation (van der Meijden & Heemskerk, 2019). Platelets are also recognized for their roles in immunothrombosis (Heestermans et al., 2022) and immune‐mediated inflammatory diseases (Scherlinger et al., 2023). Upon activation, platelets release variable classes of soluble bioactive molecules and platelet‐derived extracellular vesicles (PEVs) (van der Meijden & Heemskerk, 2019). Previous studies have shown that platelets are probably the most tunable cells regarding different biogenetic pathways for EV formation, and that the molecular cargo of PEVs, such as their proteome (Aatonen et al., 2014; Milioli et al., 2015), miRNAs (Ambrose et al., 2018) and even the content of cytoplasmic organelles like mitochondria (Brisson et al., 2017), can vary depending on receptor‐mediated activation or the cellular environment, for example, depending on the shear conditions in circulation (Tersteeg et al., 2014). Early electron microscopy (EM) analysis of activated platelets described two populations of PEVs: microvesicles and exosomes (Heijnen et al., 1999). Subsequent EM analyses of PEVs from activated platelets have revealed several morphological classes, including spherical EVs, tubular EVs and large platelet fragments in a size range of 50–1000 nm (Brisson et al., 2017; De Paoli et al., 2018) illustrating the capacity of platelets to generate heterogenous EV subpopulations (Aatonen et al., 2012; Gasecka et al., 2019; Multia et al., 2019). However, the functionality of these different PEVomes (i.e., all platelet EVs) remains poorly understood.

EVs containing the platelet and megakaryocyte specific integrin subunit CD41 (combined with CD61) constitute ∼30%–52% of all circulating EVs depending on the used analytical method (Arraud et al., 2014; Berckmans et al., 2019). Although some of these EVs may be of megakaryocyte‐origin (Flaumenhaft et al., 2010), elevated levels of circulating PEVs have been associated with pathological conditions with immune‐mediated inflammatory mechanisms, such as sepsis (French et al., 2020; Jiang et al., 2023), autoimmune conditions (lupus, rheumatoid arthritis) (Boilard et al., 2010), cardiovascular diseases (Badimon et al., 2022) and cancer (Lazar & Goldfinger, 2021). Regarding physiological functions, roles of PEVs have been assigned to coagulation and into recruitment and coordinating ‘homing’ of innate immune cells to the site of vascular injury/infection, whereas their role in coordinating adaptive immune cell functions is less explored (Boilard & Bellio, 2022).

We hypothesized that to be relevant in different physiological and pathological immune related functions, platelets must be able to control their PEV release to match the activating signal, which makes them exceptionally agile cells regarding EV‐production, and, in turn, these PEVs represent an elegant manner to further disseminate immunomodulation. Thus, we explored the possibility to selectively tune the PEV release by targeting two immunoreceptors containing the immunoreceptor tyrosine‐based activation motif (ITAM), glycoprotein VI (GPVI) and C‐type lectin‐like receptor 2 (CLEC‐2), which share the same downstream signaling, but are differently engaged in vivo (Rayes et al., 2019). Regarding inflammatory diseases, GPVI is a master regulator of PEV formation, for example, in rheumatoid arthritis in vivo in mice (Boilard et al., 2010) and is currently a novel drug target for acute ischemic stroke, atherosclerosis (Wichaiyo et al., 2022) and cancer (Garcia‐Leon et al., 2024). GPVI is activated by collagen and fibrin/fibrinogen (Alshehri et al., 2015) in a thrombus. In contrast, CLEC‐2 is a receptor for viruses such as Dengue (Sung et al., 2019) and human immunodeficiency virus‐1 (Chaipan et al., 2006), and can also be activated by podoplanin (Suzuki‐Inoue, 2018), which is relevant for cancers, such as melanoma (Suzuki‐Inoue et al., 2017). Importantly, these two ITAM‐receptors share signaling pathways which are also common for key immunoreceptors in many immune cells including myeloid, plasma dendritic, B‐ and T‐cells (Abram & Lowell, 2007; Watson et al., 2010). Thus, findings of shared or different properties of PEVs formed upon the engagement of GPVI and CLEC‐2 could be of general interest for immune‐related inflammatory functions. For this study, we employed the following specific receptor agonists: GPVI PEVs were induced by collagen related peptide (CRP‐XL) (Kehrel et al., 1998) and CLEC‐2 PEVs by snake venom toxin, rhodocytin (Watson et al., 2008). As a well‐established PEV‐inducer, we employed thrombin and collagen co‐activation to engage all platelet thrombin and collagen receptors (TC PEVs) (Aatonen et al., 2014; Keuren et al., 2005), which generates a signal relevant to, for example, cardiovascular pathologies and vascular injury. For comparison, we also isolated PEVs from unstimulated platelets (US PEVs).

To assess the immunomodulatory roles of the differentially induced PEVs by functional testing, we engaged two model systems. First, we tested if intravenously (IV) injected PEVs home in on macrophages in circulation using a zebrafish embryo model of the vertebrate reticuloendothelial system in vivo (Campbell et al., 2018; Hayashi et al., 2020; Pattipeiluhu et al., 2022). We then explored the differences between the four PEV types using an in vitro model of human macrophages (PMA‐differentiated THP‐1 cells). Next, we employed state‐of‐the‐art EV characterization methodologies, including nanoparticle tracking analysis (NTA), microfluidic resistive pulse sensing (MRPS), single‐particle interferometric reflectance imaging sensing (SP‐IRIS) and EM to profile the four PEV types according to their physicochemical characteristics. Lastly, proteomes and miRNA cargo profiles were compared to identify molecular signatures which might define the immunomodulatory capabilities of the four PEV types. Surprisingly, neither the conventional EV characterization nor the compositional analysis could predict the immunomodulatory properties of the PEVs observed in this study. Our study thus highlights the importance of functional testing beyond physicochemical characterization and omics.

## 2 MATERIALS AND METHODS

### 2.1 Induction and isolation of platelet‐derived extracellular vesicles from platelets

A workflow is given in Figure S1. Standard leukocyte‐reduced platelet concentrates, each derived from the buffy coats of four ABO RhD‐matched whole blood donations, were obtained from the Finnish Red Cross Blood Service (Helsinki, Finland) and handled anonymously, as accepted by the Finnish Supervisory Authority for Welfare and Health (Valvira, Helsinki, Finland) (Ilvonen et al., 2024). Platelet concentrates were prepared <24 h of donation and the platelets were isolated by size‐exclusion chromatography (SEC) immediately upon arrival (Aatonen et al., 2014). Platelet concentration was measured with the Beckman Coulter T‐540 haematology analyser (Beckman Coulter, USA) and adjusted to 2.5 × 10^8^ mL^−1^ with Tyrode's‐HEPES buffer supplemented with 1 mM MgCl_2_, 2 mM CaCl_2_ and 3 mM KCl (physiological cation levels), matching an average physiological platelet concentration in plasma. Platelets were activated by addition of either (i) 2 µg/mL collagen (HORM, Takeda Pharmaceuticals, Japan) and 0.2 U/mL thrombin (Enzyme Research Laboratories, UK) (TC) (Aatonen et al., 2014), (ii) 10 µg/mL CRP‐XL (CambColl Laboratories, UK) (GPVI) (Kehrel et al., 1998) or (iii) 100 nM rhodocytin (CLEC‐2) (Watson et al., 2008) for 30 min at 37°C under non‐stirring conditions. Platelets without any added agonist were treated in the same manner to obtain PEVs from unstimulated platelets. To monitor the extent of activation, changes in platelet concentration and platelet surface P‐selectin (CD62P) from platelets and PEVs and activated CD41/CD61 (PAC1) from platelets were determined with high sensitivity flow cytometry (Figure S2c). Activation time for the generation of PEVs for the comparative studies was optimized by measuring time curves of CD61+ PEVs (15, 30, 60 and 180 min) to ensure sufficient responses for all agonists and the 30‐min timepoint was chosen for further experiments (Figure S2d). After activation, platelets were sedimented at 2500 × *g* for 15 min at room temperature (RT), after which the supernatant was transferred to a fresh tube and centrifuged at 2500 × *g* for 15 min at RT to ensure removal of platelet remnants (Palviainen et al., 2020). The absence of residual platelets was verified with the haematology analyser and the supernatants were analyzed directly or used for EV isolation. For the PEV isolation, supernatant was overlaid onto a 3 mL 60% iodixanol cushion and centrifuged (Thermo Scientific Sorvall WX Ultra) at 100,000 × *g* (SW28Ti rotor, Beckman Coulter) for 3 h after which the fraction above the iodixanol cushion was collected and resuspended in 10 mL of DPBS filtered with a 0.1 µm filter unit (Millex VV, Millipore) to remove any residual iodixanol. PEVs were then concentrated with 10 kDa cut‐off ultrafiltration devices (Amicon Ultra‐15 Centrifugal Filter Unit, Millipore) to a volume of 100 µL. Single‐use aliquots were stored at −80°C and used within 3 months from isolation. The PEVs isolated in this manner comprise heterogenous subpopulations, including but not limited to microparticles and exosomes (Heijnen et al., 1999).

### 2.2 High sensitivity flow cytometry

Twenty microlitres of platelet post activation supernatants were incubated with 2.5 µL of phycoerythrin (PE)‐conjugated mouse anti‐human CD62P (clone AK‐2, BD Biosciences), 2.5 µL of PE‐conjugated CD61 (clone VI‐PL2, BD Biosciences), or 2.5 µL of fluorescein isothiocyanate (FITC)‐conjugated anti‐human CD41/CD61 (clone PAC‐1, BioLegend) for 2 h at RT in the dark. Isotype‐matched negative controls (i.e., samples incubated with mouse PE‐conjugated IgG1 or FITC‐conjugated IgM at the same concentration as the corresponding antibodies) and unlabeled EVs were used to exclude non‐specific binding of antibodies and background, respectively. After incubation, the samples were diluted 1:10 with 10 mM Hepes buffer containing 140 mM NaCl, pH 7.2 (NH buffer) that was filtered through 0.1 µm filter unit. All samples were analyzed on an Apogee A50‐Micro flow cytometer (Apogee Flow Systems Ltd. UK) at a flow rate of 1.5 µL/min. The flow rate was calibrated using Apogee bead mix (Apogee Flow Systems Ltd. UK) by measuring the concentration of 110 nm FITC‐labeled PS beads and comparing the measured concentration to the reference value given by the manufacturer. Each sample was measured for 90 s, with triggering at 405 nm (side scatter). The data were analyzed with FlowJo (v10.7.1; FlowJo). A Rosetta Calibration system (Exometry, The Netherlands) was used to calibrate the light scatter signal and to correlate it to particle diameter (van der Pol et al., 2018). Based on the calibration, size gates were set for PEVs (200–1000 nm), cell remnants (1000–2000 nm) and platelets (2000–3000 nm) (Figure S2b). Gates for fluorescent signal were set based on isotype controls. Events exceeding the fluorescence threshold were determined as positive. The presented concentrations represent the number of detected events adjusted for the overall sample dilution, flow rate and measurement duration. The dilution buffer and free antibody in buffer were used as additional controls according to MIFlowCyt guidelines (Welsh et al., 2020).

### 2.3 NTA

Particle concentration and size distribution were measured with NTA from six biological replicates (24 donors) using a Nanosight instrument LM14 (Malvern Instruments, UK) equipped with a violet laser (405 nm, 70 mW) and a sCMOS camera (Hamamatsu Photonics, Japan). The samples were diluted in 0.1 µm filtered DPBS to obtain 40–100 particles/frame, and five 30 s videos were recorded with camera level 14. The data were analyzed using NTA 3.0 software (Malvern Panalytical, UK) with detection threshold at 4 and screen gain at 10. Statistical analysis was performed using Prism 9 software (GraphPad, USA). Statistical significance was determined using the Kruskal–Wallis test (*p* ≤ 0.05).

### 2.4 MRPS

Particle concentration and size distribution were measured with MRPS using a Spectradyne nCS1 instrument (Spectradyne, USA) equipped with C300 polydimethylsiloxane cartridges to cover a size range of 50–300 nm in vesicle diameter. Samples were diluted in filtered 1% Tween‐20 in DPBS to obtain 10^7^–10^10^ particles/mL and measured immediately with a sample volume of 4 µL. Data were collected for 300 s or until a minimum of 1000 particle transition events were counted per analysis. Data were pre‐processed with the nCS1 Data Analyzer (Spectradyne) by combining events from two technical replicates, by removing electrical noise and buffer background and by applying cartridge‐specific filters to exclude false‐positive signals based on transit time versus diameter, peak symmetry, and signal to noise ratio. The results of data‐filtering were graphed in 2D scattergrams and inspected for each run. Statistical analysis was performed using Prism 9 software (GraphPad). Statistical significance was determined using the Kruskal–Wallis test (*p* ≤ 0.05).

### 2.5 Transmission electron microscopy (TEM)

PEVs were loaded on 200 mesh pioloform carbon coated glow discharged copper grids, fixed with 2% PFA, stained with 2% neutral uranyl acetate, embedded in methyl cellulose uranyl acetate mixture (1.8%/0.4%) and viewed with Tecnai 12 (FEI Company, The Netherlands) at 80 kV.

### 2.6 Scanning electron microscopy (SEM)

Platelet activation was stopped after 20 min by adding 2% glutaraldehyde in 0.1 M Na‐cacodylate buffer (NaCaC buffer), pH 7.4. The platelets were then transferred to poly‐L‐lysine coated coverslips, and fixation was continued for 20 min at RT. After fixation, the coverslips were rinsed twice with NaCaC‐buffer for 3 min each, osmicated for 60 min at RT in 1% OsO4 dissolved in 0.1 M NaCaC‐buffer, dehydrated in a graded series of ethanol/water (50%–100%, v/v) and critical point dried. The samples were then platinum‐sputtered and examined using a Quanta 250 Field Emission Gun (FEI Company, USA) scanning electron microscope at 3 kV.

### 2.7 Single particle interferometric reflectance imaging sensor (SP‐IRIS)

PEV samples were analyzed with the ExoView Plasma Tetraspanin kit and an ExoView R100 (NanoView Biosciences, USA). The samples were diluted with incubation buffer to a desired concentration (1–3 × 10^8^ particles in total) based on NTA measurement. Thirty‐five microlitres of sample was added directly on the chip and incubated at RT for 16 h. The samples were then stained with a cocktail of fluorescently labeled antibodies containing anti‐human CD81 (JS‐81, CF555), anti‐human CD63 (H5C6, CF647) and anti‐human CD9 (HI9a, CF488), washed, dried and scanned in the Exoview scanner for interferometric reflectance imaging and fluorescent detection. The data were analyzed using the NanoViewer analysis software (NanoView Biosciences, USA), using fluorescent cutoffs as follows: 250 AU for the CF555 channel, 480 AU for the CF488 channel and 269 for the CF647 channel. Statistical analysis was performed using Prism 9 software (GraphPad). Statistical significance was determined using two‐way ANOVA (*p* ≤ 0.05).

### 2.8 Proteomics

PEV samples (total of 1 × 10^10^ particles) were first dried in a vacuum concentrator (Heto VR‐maxi ST, Heto Lab Equipment), and resuspended in 50 nM ammonium bicarbonate buffer (AMBIC), pH 7.8, supplemented with 0.1% RapiGest SF (Waters, USA). Protein concentrations of the samples were determined with a standard BCA protein assay (Thermo Fisher Scientific) and 4 µg of each sample was pipetted into a final volume of 50 µL in AMBIC for the tryptic peptide preparation. Tryptic peptides were prepared using an in‐solution tryptic digestion method, according to the following protocol: 5 min denaturation at 95°C, 30 min disulfide reduction with 5 mM Tris (2‐carboxyethyl) phosphine hydrochloride (TCEP) solution at 70°C, followed by 30 min alkylation at RT in the dark with 5 mM iodoacetamide. Sequencing grade trypsin (Promega, USA) was added 1:50 enzyme:protein for 2 h at 37°C, and again at the same concentration for overnight incubation at 37°C. After overnight digestion, formic acid was added to a final concentration 0.1%, followed by incubation at 37°C for 45 min to remove RapiGest SF, after which the samples were centrifugated at 13,000 rpm for 15 min to remove residual debris and transferred into 250 µL autosampler microvials (Thermo Fisher Scientific). Samples were then loaded into an Easy‐nLC 1200 (Thermo Fisher Scientific) coupled to an Orbitrap Fusion mass spectrometer (Thermo Fisher Scientific). Peptides (100 ng) were separated using Acclaim PepMap C18 columns (2 µm, 100 Å, 75 mm, 15 cm; Thermo Fisher Scientific). The peptides were loaded in buffer A (5% acetonitrile and 0.1% formic acid) and eluted with a 1 h linear gradient from 10% to 40% buffer B (80% acetonitrile and 0.1% formic acid). Three biological replicates (12 donors), each in three technical replicates, were sequentially injected with two 15 min wash runs and a 1 h blank run alternated between distinct ‘treatments.’ Mass spectra were acquired using a cycle time data‐dependent method with an automatic switch between full MS and MS/MS (MS2) scans every 3 s. The Orbitrap analyser parameters for the full MS scan were resolution of 120,000, mass range of 350 to 1800 m/z and standard AGC target, whereas those for MS2 spectra acquisition were resolution of 30,000, AGC target of 5 × 10^4^ ions, with an isolation window of 2 m/z and dynamic exclusion of 30 s. Column chromatographic performance was routinely monitored with intermittent injections of 50 fmol of a commercially available BSA peptide mix (Bruker Corporation, USA) and by evaluating double‐wash runs for carry‐over peptides.

Protein identification and quantification were carried out with MaxQuant (Tyanova et al., 2016) software package v. 1.6.17.0, with the UniProtKB human FASTA file containing >86,000 entries with 245 commonly observed contaminant and all reverse sequences. Technical replicates (*n* = 3) of each sample were matched between runs to transfer identifications between replicates. All other parameters were used in their default settings. Perseus data analysis software (Tyanova et al., 2016) v.1.6.14.0 was used for differential abundance analysis and hierarchical clustering. Abundance values were log2 transformed, protein identifications classified as being only identified by site, and reverse sequences and potential contaminants were filtered out from the data frame. Additionally, identifications with zero intensity values in all biological replicates were excluded from the comparison. Missing intensity values of were imputed from normal distribution, and abundance intensities were median normalized. Statistical analysis was performed using Prism 9 software (GraphPad). Statistical significance was determined using the Kruskal–Wallis test (*p* ≤ 0.05).

### 2.9 Proximity extension assay (PEA)

The inflammation‐related protein cargo of PEVs (n = 3; 12 donors) was analyzed with the PEA technology (Assarsson et al., 2014) using Olink Inflammation panel (De Paoli et al., 2018). The protein concentration of PEVs were measured with DC assay (Bio‐Rad) according to manufacturer's instructions using BSA as a standard. Samples were normalized to 90 µg/mL prior the analysis. Data are expressed as normalized protein expression (NPX) values on a log2 scale whereby a higher NPX correlates with higher protein expression. Data normalization (against extension control and inter plate control) was performed to minimize both intra‐and inter‐assay variation. Proteins containing NPX values > 50% below the assay's limit of detection (LOD) were excluded from the analysis. Values below LOD were replaced by LOD/2 and linearized expression of the log2 scale was used for statistical analysis. Statistical analysis was performed using Prism 9 software (GraphPad). Statistical significance was determined using the Kruskal–Wallis test (*p* ≤ 0.05).

### 2.10 Small RNA analysis

PEV samples (total of 1 × 10^10^ particles, 100 µL) from four biological replicates (16 donors) were diluted to 500 µL with DPBS, and then pelleted at 110,000 g for 90 min at 4°C by an Optima MAX‐XP ultracentrifuge with a TLA‐55 rotor, k factor 81.3 (Beckman Coulter). RNA was isolated from the pelleted samples with a miRNeasy micro kit (Qiagen) following the manufacturer's instructions and stored at −80°C for further analysis. The quantity and quality of RNA in the samples were evaluated with the Qubit RNA HS Assay kit (Thermo Ficher Scientific), and Agilent RNA 6000 Pico Chips (Agilent Technologies).

Small RNA sequencing libraries were prepared following the protocol included in the kit NEXTflex™ Small RNA‐Seq kit v3 (Bioo Scientific Co). Briefly, total RNA of each sample (between 200 and 10,000 pg) was incubated for 2 min at 70°C, then 3′ 4N adenylated adapter (adapter dilution 1/4) and ligase enzyme were added, and ligation was conducted by incubation of this mix at overnight at 20°C. After excess 3′ adapter removal, 5´‐adapter was added alongside with ligase enzyme and the mix was incubated at 20°C for 1 h. The ligation product was used for the reverse transcription with the M‐MuLV Reverse Transcriptase in a thermocycler for 30 min at 42°C and 10 min 90°C. Next, enrichment of the cDNA was performed using PCR cycling: 2 min at 95°C; 22–25 cycles of 20 s at 95°C, 30 s at 60°C and 15 s at 72°C; a final elongation of 2 min at 72°C and pause at 4°C. PCR products were resolved on 8% Novex TBE PAGE gels (Thermo Fisher Scientific), and one band between 150 and 300 bp was cut from the gel. Small RNAs were extracted from the polyacrylamide gel using an adapted protocol, in which DNA was eluted from the gel slices in nuclease free water overnight at RT. Afterwards, libraries were quantified using a Qubit dsDNA HS DNA Kit (Thermo Fischer Scientific) and visualized on an Agilent 2100 Bioanalyzer using an Agilent High Sensitivity DNA Kit (Agilent Technologies). Libraries were sequenced in a HiSeq2500 (Illumina) by at least 12 million 51‐nucleotide reads. Once raw counts were extracted for all the samples, lowly expressed genes were filtered out by running a proportion test, as implemented in the NOISeq package (Tarazona et al., 2015). Normalization and differential expression analysis were performed by using the DESeq2 algorithm (Love et al., 2014). To correct the *p*‐values for multiple testing, the False Discovery Rate (FDR) method was applied. Genes were considered differentially expressed if adjusted *p*‐value < 0.05. miRNAs target prediction was performed using the multiMiR Bioconductor package (Ru et al., 2014). In particular, we focused our prediction on databases of validated targets, including mirecords (Xiao et al., 2009), mirtarbase (Huang et al., 2021) and tarbase (Sethupathy et al., 2006). The functional profiling of the miRNA target genes was performed by using the clusterProfiler Bioconductor package (Wu et al., 2021).

### 2.11 In vivo functional assay

#### 2.11.1 Zebrafish lines and maintenance

All zebrafish (*Danio rerio*) work was performed in accordance with Danish and EU legislation under permit 2022‐15‐0202‐00169 from the Danish Animal Ethics Council. Zebrafish were maintained under standard conditions (temperature 28°C, pH 7.2 and conductivity 700 µS) in recirculating housing systems on a 14 h light—10 h darkness cycle and fed three times daily. Embryos were generated by natural crosses and maintained in E3 medium (5 mM NaCl, 0.17 mM KCl, 0.33 mM CaCl_2_, 0.33 mM MgSO4, 10^−5^% (w/w) methylene blue, 2 mM HEPES, pH 7.2) at 28°C. Experiments were performed on <5 days post‐fertilization (dpf) embryos from established transgenic lines; *Tg(mpeg1:mCherry)^gl23^* as a reporter line for embryonic macrophages (Ellett et al., 2011), and *Tg(tnfa:EGFP‐F)^ump5^* for transcriptional activation of *tnfa* (Nguyen‐Chi et al., 2015). All fish lines and wild type (AB) used in this study were originally obtained from the European Zebrafish Resource Centre (Germany).

#### 2.11.2 Intravenous microinjections of PEVs

All microinjection experiments were performed as previously described (Hayashi et al., 2020). Briefly, zebrafish embryos (2 dpf) were dechorionated, anesthetized in E3 medium with 0.016% (w/v) buffered tricaine (3‐amino benzoic acid ethyl ester; Sigma‐Aldrich), and embedded in 0.8% (w/v) low‐melting‐point agarose (BioReagent; Sigma‐Aldrich). They were then IV injected via the common cardinal vein (Benard et al., 2012) with 3 nL of PEVs along with loading buffer (sterile‐filtered, endotoxin‐free phenol red solution in DPBS; BioReagent, Sigma‐Aldrich) giving a nominal dosage range of 10^5^–10^6^ particles per bolus estimated by NTA. Injected embryos were imaged in vivo without further handling.

#### 2.11.3 Image acquisition, processing and analysis

As described previously in more detail (Hayashi et al., 2020; Mohammad‐Beigi et al., 2020), all images were acquired using a Zeiss LSM 780 upright confocal microscope (Carl Zeiss Inc., Germany) with excitation lasers at 488 nm (EGFP), 568 nm (mCherry) and 633 nm (PEVs labeled with CellTrace^TM^ Far Red dye). The objective lens used was W Plan‐Apochromat 40×/1.0 DIC M27 (Carl Zeiss). All images at the designated tissue area were acquired at 2‐µm intervals ensuring optical section overlap in each fluorescence channel to construct *z*‐stack images and presented as the maximum intensity *z*‐stack projections using Fiji/ImageJ (Schindelin et al., 2012). Bright‐field image is shown as a single optical slice at an arbitrary position. PEV colocalization with macrophages was determined using a 3D mask approach based on thresholding on the fluorescence intensity of the macrophage reporter protein and the ‘Convert to Mask’ tool in Fiji/ImageJ as described previously (Hayashi et al., 2020). Briefly, for quantification of the PEV fluorescence overlapping with macrophages, the binary mask created was applied by inverting LUT to the channel for PEV fluorescence. This procedure was performed for all optical slices in a stacked image to exclude PEV fluorescence colocalized in the *x, y* space but not in the *z* axis. All images were processed as the maximum intensity *z*‐stack projections and analyzed using Fiji/ImageJ. ‘Sequestered PEVs’ were defined by thresholding of the PEV signals typically picking only immobilized clusters associated with cells. For quantification of the relative contribution of macrophages to overall PEV sequestration, the area of macrophage masked PEV signal (>threshold) was divided by the area of total PEV signal (>threshold). Since this analysis was critically affected in minor cases where macrophages moved in and out of the field of view, we excluded embryos by applying a criterion that the over‐time average of macrophage‐masked PEV area should be >25 µm^2^.

### 2.12 In vitro functional assays

#### 2.12.1 Cell culture and differentiation of THP‐1 cells

THP‐1 cells (ATCC TIB‐202), a human monocytic cell line, were grown in culture medium consisting of RPMI 1640 (Euroclone, Italy) supplemented with 10% EV‐depleted foetal bovine serum (FBS, Gibco), 100 U/mL penicillin (Gibco), 100 µg/mL streptomycin (Gibco), 2 mM L‐glutamine (Gibco) and 25 mM HEPES (pH 7.4), referred hereafter to as the medium. EV depletion was performed by polyethylene glycol precipitation (Kyykallio et al., 2022). THP‐1 cells were plated to a 24‐well plate at a concentration of 2 × 10^5^ cells/well and incubated for 48 h in 1 mL/well of the medium supplemented with 50 nM phorbol 12‐myristate 13‐acetate (PMA) (Sigma‐Aldrich) for differentiation into macrophages. For PEV uptake experiments (Section 2.12.4), THP‐1 cells were plated at a concentration of 0.5 × 10^5^ cells/well on a 96‐well black PhenoPlate (Revvity, Finland) coated with 5 µg/cm^2^ of fibronectin (Roche, Switzerland) and incubated for 48 h in 100 µL/well of the medium supplemented with 50 nM PMA. Viability of the cells was analyzed by Trypan Blue exclusion test using the Countess Automated cell Counter (Thermo Fischer Scientific). After differentiation, cells were washed with PBS and allowed to rest for 24 h in fresh medium before administration of PEVs.

#### 2.12.2 Cytokine analysis with ProcartaPlex immunoassay

PMA‐differentiated THP‐1 cells were treated either with GPVI PEVs, CLEC‐2 PEVs, TC PEVs, or US PEVs for 6 and 24 h in a 24‐well plate (*n* = 4). For each treatment, a dose of 2.5 × 10^9^ PEVs/well (2 × 10^5^ cells) was used, as determined previously (Maaninka et al., 2023). As a control, four wells were grown without PEV addition (mock treatment). After the incubation, the conditioned media was collected, centrifuged at 500 × *g* for 5 min at 4°C to remove cellular debris, and stored at −80°C. Cytokine secretome was analyzed using the Cytokine & Chemokine 34‐Plex Human ProcartaPlex Panel 1A (Invitrogen) and measured with the xMAP Luminex system (Bio‐Rad) according to the manufacturer's instructions. The assay included granulocyte‐macrophage colony‐stimulating factor (GM‐CSF), monocyte chemoattractant protein (MCP) 1, macrophage inflammatory protein (MIP) 1α, MIP‐1β, RANTES (regulated on activation, normal T cell expressed and secreted), stromal cell‐derived factor 1 (SDF‐1α), gamma‐induced protein 10 (IP‐10), eotaxin, IFN‐γ, interleukin (IL) IL‐1α, IL‐1β, IL‐1RA, IL‐2, IL‐4, IL‐5, IL‐6, IL‐7. IL‐8, IL‐9, IL‐10, IL‐12p70, IL‐13, IL‐15, IL‐17A, IL‐18, IL‐21, IL‐22, IL‐23, IL‐27, IL‐31, tumor necrosis factor α (TNFα), TNFβ, interferon (IFN) α, and chemokine (C‐X‐C motif) ligand (CXCL) 1 (GRO‐α). The samples were pre‐diluted 1:2 with RPMI media. Standard curves for each protein were generated with a 5‐parameter logistic (5‐PL) algorithm, reporting values for both median fluorescence intensity (MFI) corrected with background, and concentration. Proteins with concentrations within the detection range with all biological replicates in at least one sample group were included for further analysis. Log‐transformed MFIs were used for statistical analysis demonstrating advantages for including low and high abundant analytes (Breen et al., 2016). Statistical analysis was performed using Prism 9 software (GraphPad). Statistical significance was determined using the Kruskal–Wallis test (*p* ≤ 0.05).

#### 2.12.3 PEV labeling for uptake assay

PEVs in the range of 1.4–7.7 × 10^11^ mL^−1^ were incubated with 20 µM CellTrace^TM^ Far Red (Invitrogen) for 30 min at 37°C in the dark, with mixing at 15 min. After incubation, unbound label was immediately removed by Izon qEV single Gen2 70 nm columns (Izon Science), with 0.1–0.15 mL of labeled PEVs loaded per column. After discarding the first 0.65 mL of flow‐through, five 0.15 mL fractions were collected with an Automatic Fraction Collector (Izon Science) using 0.1 µm filtered dPBS as elution buffer. The EV fractions were pooled and concentrated with 10 kDa cut‐off ultrafiltration devices (Amicon Ultra‐4 Centrifugal Filter Unit, Millipore) to a volume of 100 µL. Particle concentration was measured with NTA, and the sample was stored in protein LoBind tubes (Eppendorf) at 4°C until used for the cell experiment the next day. Buffer (dPBS filtered with 0.1 µm filter unit) with 20 µM CellTrace^TM^ Far Red was prepared identically to the PEV samples and used as a mock control.

#### 2.12.4 PEV uptake by high content imaging

To study the uptake of PEVs by macrophages, THP‐1 cells (0.5 × 10^5^ cells/well in 100 µL of the medium) were grown on a 96‐well PhenoPlate and differentiated into macrophages as described above. Cells were washed with PBS, incubated with 0.25 µM CellTrace^TM^ CFSE (Thermo Fisher Scientific) in pre‐warmed PBS for 20 min at 37°C, washed again, and allowed to rest in fresh medium for 30 min. Cells were then treated with a dose of 2.5 × 10^8^–5.0 × 10^8^ CellTracet^TM^ Far Red‐labeled PEVs or with the mock control. The media was removed at 3, 6, 9 and 12 h, and the cells were washed and fixed for 15 min with 4% paraformaldehyde. Cells were washed twice with PBS, permeabilized with 0.5% Triton‐X‐100 (Thermo Fisher Scientific), and blocked with PBS supplemented with 10% FBS (Gibco) for 15 min. The cells were washed twice with PBS and stained with mouse anti‐human CD18 primary antibody (Santa Cruz, clone sc‐8420) at 1:100 dilution in PBS overnight at 4°C to visualize the cell membrane. The next day, cells were washed twice with PBS and stained with anti‐mouse Alexa Fluor 555 secondary antibody (Invitrogen) at RT for 2 h in the dark. The wells were washed with PBS and imaged with the PerkinElmer Opera Phenix High Content Screening System with 20× NA 1.0 water immersion objective at the High Content Imaging unit of the Finnish Institute for Molecular Medicine (FIMM, Helsinki, Finland). Image analysis was done with Cell Profiler (v. 4.2.5) (Stirling et al., 2021) and at least 700 cells per sample were counted.

## 3 RESULTS

### 3.1 Platelets tune EVs for immunomodulatory functions

We employed receptor‐specific agonists to study how platelets tune EVs for immune functions. We compared activation by two immunoreceptors, GPVI and CLEC‐2, which share similar downstream signaling in platelets that is analogous to immunoreceptors in leukocytes. GPVI receptors were activated with CRP‐XL (GPVI PEVs), and CLEC‐2 receptors with rhodocytin (CLEC‐2 PEVs). Thrombin and collagen receptors were collectively activated by a co‐stimulus (TC PEVs). We also isolated PEVs from unstimulated platelets to which no exogenous activator was added (US PEVs). Following careful removal of residual platelets after activation or mock treatment, PEVs were isolated using iodixanol cushion ultracentrifugation yielding heterogeneous mixtures of EV subpopulations. The workflow for platelet isolation, activation, and PEV isolation is detailed in Figure S1 and comparative data of platelet activation by the agonists are given in Figure S2.

**FIGURE 1:**
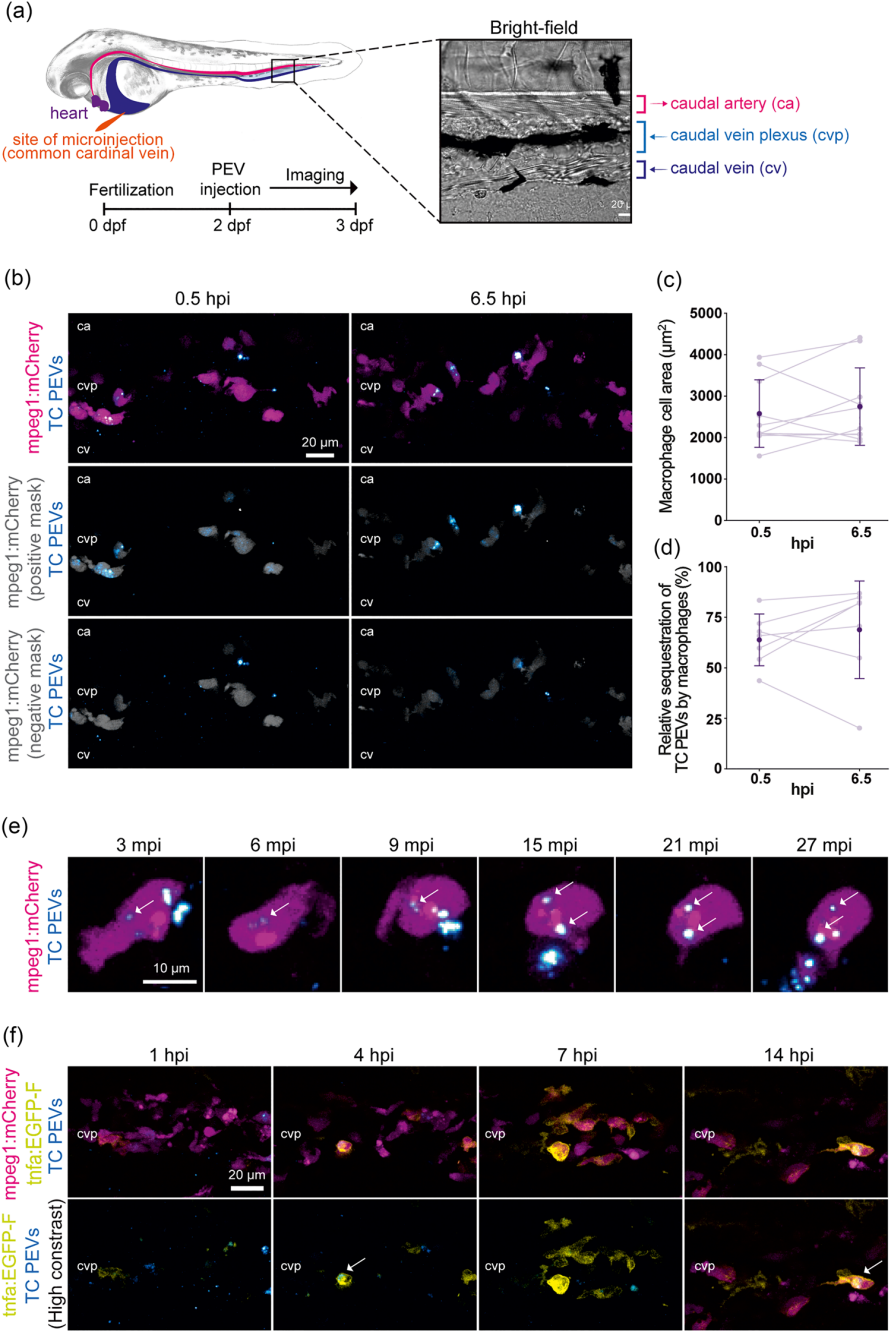
Interaction of macrophages with PEVs in vivo. Fluorescently labeled PEVs from platelets activated by TC co‐stimulation were intravenously injected into zebrafish embryos at 2 dpf and live‐imaged. (a) Left panel shows a simplified schematic of the site of microinjection and the main blood vessels including the ca, cv and cvp along with a representative BF image of the tissue area imaged. Right panel shows an overview of the experimental setup. (b)–(d) *Tg(mpeg1:mCherry)* embryos were imaged every 20 min from 0.5 to 6.5 hpi. Representative images show the total TC PEV signals (cyan) and macrophages (magenta) at two time points (top panel). The two lower panels display the same images after segmentation by a macrophage‐specific mask (gray) to determine PEV colocalization with macrophage reporters in spatial *x‐y‐z* dimensions. The panel with the ‘positive mask’ shows only TC PEV signals colocalizing with macrophages, while the ‘negative mask’ panel reveals those that are excluded, such as associations with cells other than macrophages. Scale bar = 20 µm. (c)–(d) Image analysis. trendlines represent individual embryos in light purple (*n* = 10) with mean ± SEM in dark purple (*n* = 7, after excluding embryos where macrophages were moving in and out of the field of view). Relative TC PEV sequestration by macrophages (d) is the area ratio of the macrophage‐masked TC PEVs (b, middle panel) to the total TC PEVs (b, top panel). (e) *Tg(mpeg1:mCherry)* embryos were injected and imaged in a time‐lapse sequence from 3 to 27  mpi with a focus on the interactions of TC PEVs (cyan) with a single macrophage (magenta). Arrows indicate TC PEVs that colocalize with the macrophage reporter. Scale bar = 10 µm. (f) *Tg(mpeg1:mCherry); Tg(tnfa:EGFP‐F)* embryos at 2 dpf were injected with fluorescently labeled TC PEVs and imaged every 30 min from 1 to 14 hpi. Representative images show transcriptional activation of *tumor necrosis factor‐alpha* (*tnfa*, yellow) over time in macrophages (magenta) with or without sequestered TC PEVs (cyan). Arrows indicate examples of macrophages that had sequestered TC PEVs and induced *tnfa*. Scale bar = 20 µm. Anterior left, dorsal top. BF, bright‐field; ca, caudal artery; cv, caudal vein; cvp, CV plexus; dpf, days post‐fertilization; hpi, hours post‐injection; mpi, minutes post‐injection; PEVs, platelet‐derived extracellular vesicles; TC, thrombin and collagen.

We first explored how PEVs circulate in the blood and interact with cells in vivo using a zebrafish model. For live imaging, transgenic embryos (2 dpf) were IV injected with CellTrace^TM^ Far Red‐labeled PEVs. The transgene *Tg(mpeg1:mCherry)^gl23^* drives constitutive expression of the fluorescent reporter protein mCherry in all embryonic macrophages, and thereby intracellular co‐localization of the two fluorophores indicates accumulation of PEVs in macrophages. Preliminary screening revealed that only the GPVI and TC PEVs of the four PEV types had consistently sufficient concentrations to enable quantitative image analysis. Therefore, as TC is a well‐established inducer of PEVs that also encompasses GPVI activation, we focused on the TC PEVs to ask whether macrophages selectively take up PEVs. Roughly 10^5^–10^6^ particles (per bolus, as estimated by NTA) were microinjected into systemic circulation via the common cardinal vein that returns peripheral blood into the heart. PEV accumulation was only observed in the caudal vein (CV) and the CV plexus (CVP) that represent the reticuloendothelial system (Figure 1a, with a bright‐field image of the tissue of interest). PEVs homed in on blood‐resident macrophages already at 0.5 h and this pattern remained for 6 h (Figure 1b, Movie 1). Image analysis supported the observation that PEVs primarily accumulated in macrophages reaching ∼70% of total sequestration, while there was no change in the number of macrophages observed in‐frame over time (Figure 1c,d). The same homing pattern was observed for the GPVI PEVs (Figure S3, Movie 2). The time‐lapse sequence starting from 3 mpi depicts the interaction of a single macrophage with TC PEVs, some of which are clearly co‐localized with the macrophage reporter spatially and temporally (Figure 1e). It should also be noted that the injected PEVs were rarely detectable in the circulation, indicating rapid sequestration from the bloodstream. To further study the functional role of macrophage‐targeting PEVs, we traced the transcriptional activation of the tumor necrosis factor alpha gene (*tnfa*) using a double transgenic combination of *Tg(mpeg1:mCherry)^gl23^* and *Tg(tnfa:EGFP‐F)^ump5^* lines. Within 3 h of the TC PEV injection, macrophages started to induce *tnfa*, indicating their polarization towards a pro‐inflammatory phenotype (Figure 1f, Movie 3).

**FIGURE 2:**
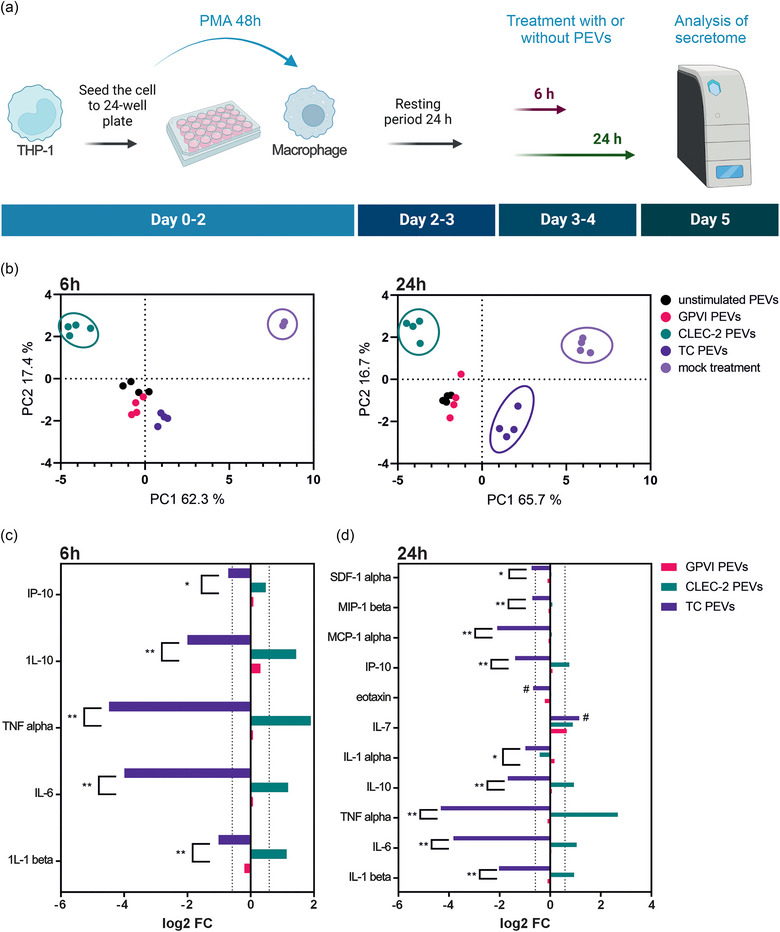
Changes in the macrophage cytokine secretomes induced by the different PEV types. (a) Flow chart of the experiment. THP‐1 cells were differentiated into macrophages by 48 h incubation with 50 nM PMA. After a resting period of 24 h, macrophages were treated with the different PEV types (*n* = 4; biological replicates representing 16 donors) or left untreated for 6 or 24 h, after which the media was collected, processed, and analyzed for 34 cytokines using a Luminex inflammation panel. Figure created with BioRender.com. (b) Principal component analysis of the cytokine secretomes of macrophages at 6 and 24 h. Secretomes from the mock‐treated macrophages remained distinct from the PEV‐induced secretomes at both time points. The CLEC‐2 PEV‐induced secretome separated from the other PEV‐induced secretomes at 6 h, and the TC PEV‐induced secretome separated from the other PEV‐induced secretomes at 24 h. The secretomes from the macrophages treated with the GPVI or US PEVs remained grouped together. (c) Bar graph showing fold changes (>1.5 fold change, 0.585 log2 FC) in the fluorescence intensities of cytokines from the GPVI, CLEC‐2 and TC PEV‐treated macrophages compared to the cytokines from the macrophages treated with the US PEVs at 6 h. Statistical differences between the GPVI, CLEC‐2 or TC PEV‐induced secretomes are marked with asterisks (^*^ *p* ≤ 0.05, ^**^ *p* ≤ 0.01, Friedman test), and the differences between the GPVI, CLEC‐2 or TC PEV‐induced secretomes compared to the secretome induced by the US PEVs are marked with a pound sign (#). (d) Bar graph showing fold changes (>1.5 fold change, 0.585 log2 FC) in the fluorescence intensities of cytokines induced by the GPVI, CLEC‐2, or TC PEV‐treated macrophages compared to the cytokines from the macrophages treated with the US PEVs at 24 h. Statistical differences between the cytokines induced by the GPVI, CLEC‐2 or TC PEVs are marked with asterisks (^*^ *p* ≤ 0.05, ^**^ *p* ≤ 0.01, Friedman test), and the differences between the GPVI, CLEC‐2 or TC PEV‐induced secretomes compared to the secretome induced by the US PEVs are marked with a pound sign (#). PEVs, platelet‐derived extracellular vesicles.

To further elucidate the immunomodulatory effects of the PEVs on macrophages, we differentiated human THP‐1 cells with PMA into macrophages, incubated them with the PEVs and compared macrophage cytokine secretomes at two timepoints (Figure 2a). Macrophages were activated with a dose of PEVs previously optimized with primary human macrophages (Maaninka et al., 2023). The proteins with concentrations in the detection range of the assay (with all biological replicates in at least one PEV type) were included in the analysis, whereby 16 out of 34 cytokines were analyzed for statistical significance (Table S2). Treatment of macrophages with the different PEV types induced distinct secretomes both at 6 and 24 h, which were disparate from the control secretomes of the macrophages cultured in the absence of PEVs (Figure 2b). Notably, the secretomes of macrophages treated with the CLEC‐2 PEVs were distinguishable already at the 6 h time point compared to the other PEV types (Figure 2b). While the secretomes induced by the GPVI, TC, and US PEV types differed from the secretome of the untreated macrophages already at 6 h, distinct profiles among these PEV types emerged at 24 h, with the separation of the TC PEV‐induced secretome from the GPVI and US PEV‐induced secretomes. This data indicated that both the kinetics and the profiles induced by the CLEC‐2 and GPVI PEVs were different from each other and from the TC PEVs.

Further comparison of the secretomes induced by the PEVs from activated platelets (GPVI, CLEC‐2 and TC) with that of the US PEVs showed statistically significant quantitative changes in the cytokines. Five out of the 16 cytokines at the 6‐h time point (Figure 2c), and 11 out of the 16 at the 24‐h time point (Figure 2d) were differentially secreted after the treatment with the GPVI, CLEC‐2 or TC PEVs when compared to the cytokines induced by the US PEVs. At 6 h, the TC PEVs downregulated five macrophage cytokines (IP‐10, IL‐10, TNFα, IL‐6 and IL‐1β), whereas the CLEC‐2 PEVs (but only slightly the GPVI PEVs, excluding IL‐1β) upregulated these cytokines when compared to the macrophages treated with the US PEVs (Figure 2c). The same trend continued at the 24‐h time point with more cytokines, when the TC PEVs downregulated 10 cytokines (SDF‐1α, MIP‐1β, MCP‐1α, IP‐10, eotaxin, IL‐1α, IL‐10, TNFα, IL‐6 and IL‐1β) compared to the treatment with the US PEVs. Interestingly, at the 24‐h time point, IL‐7, a proliferation cytokine for lymphoid progenitor cells, was upregulated in macrophages by all the PEVs from the GPVI‐, CLEC‐2‐ and TC‐activated platelets (Figure 2d) when compared to the IL‐7 levels induced by the treatment with the US PEVs. It is noteworthy that all the PEV types induced TNFα secretion in comparison to the untreated macrophages, which supports the in vivo observation of the induction of *tnfa* in embryonic zebrafish macrophages by the circulating PEVs. To summarize the secretome results, clearly distinct macrophage cytokine profiles were induced by the CLEC‐2 and TC PEVs. These profiles were also disparate from the GPVI‐induced secretome, which was surprisingly similar to the one induced by the US PEVs.

Since the uptake of PEVs into macrophages in vivo could not be quantitated for all the PEV types, we next wanted to ensure that the functional differences seen in the THP‐1 cells were not caused by variable uptake. We investigated the uptake and cellular localization of PEVs (cytoplasm/membrane) by high content imaging at four time points over 12 h. A previously optimized number of CellTrace^TM^ Far Red‐labeled PEVs (2.5 × 10^9^–5 × 10^9^ particles/well as determined by NTA) was incubated with the PMA‐differentiated THP‐1 cells. High‐content imaging data confirmed that, in comparison to the mock control, all the four PEV types accumulated into the cytoplasm during the 12 h (Figure S4). A trend towards increased uptake of the agonist‐induced PEVs was seen compared to the US PEVs, although the difference was not statistically significant (Figure S4a). This supports the notion that the PEV‐induced functional differences in the THP‐1 cell secretomes (Figure 2b–d) were not caused by differential uptake kinetics.

### 3.2 Only few differences can be detected between the differentially induced PEVs by basic EV characterization

To identify causes for their differential functionality, the four PEV types were analyzed by physicochemical methods commonly used to characterize EVs. EM showed no notable morphological differences between the PEV types (Figure S5a). Next, particle concentration, size distribution and surface markers of the PEVs were characterized with three orthogonal single‐particle methods (Figure S5b–c). The biggest difference among the PEV‐inducing conditions was the yield. GPVI activation induced the highest particle yield (1.9 × 10^9^) per 2.5 × 10^8^ platelets/mL, followed by TC (1.7 × 10^9^) and CLEC‐2 (4.1 × 10^8^) activation, all surpassing the number of particles from unstimulated platelets (2.1 × 10^8^) when measured by NTA (Figure S5b).

The same order of potency was also detected with MRPS, but with less differences (Figure S5b). Notably, the yields from the GPVI and TC activations were similar, but despite the overlap in the GPVI and CLEC‐2 signaling pathways, CLEC‐2 was again a less efficient activator than GPVI. Substantial differences were also evident in the donors’ responsiveness to generate PEVs as judged by the large standard deviations of the particle counts (Figure S5b). Finally, the same order of potency was also seen with anti‐human CD61 or anti‐human CD62P labeling with direct analysis of the platelet activation samples (without PEV isolation) in high sensitivity flow cytometry (Figure S2d).

Next, we explored whether the activation route influenced the size distribution of the PEVs. The PEV samples were analyzed using NTA for particles ranging from 70–1000 nm, MRPS for particles within the range of 50–250 nm, and SP‐IRIS to identify CD41‐positive particles in the range of 50–200 nm (Figure S5c). Particles were sorted into bins and quantitated. The mean and mode diameters (nm ± SD) are summarized in Figure S5 table. The orthogonal particle analyses revealed that, ultimately, no significant differences could be observed in the size distribution profiles, regardless of the agonist employed for platelet activation. Only a slightly larger mean particle diameter was detected in the TC‐induced samples compared to the others, when analyzed with NTA and SP‐IRIS (CD41‐captured PEVs), but this observation was not replicated by MRPS.

Next, the subpopulation heterogeneity was inspected by SP‐IRIS. Antibodies against three common EV tetraspanins, CD9, CD63 and CD81, were used to capture PEVs in addition to the platelet‐specific antibody against CD41. The highest number of PEVs was captured with CD9 (range 1.5 × 10^7^–5.8 × 10^7^ particles), and lowest with CD81 (4.6 × 10^5^–1.2 × 10^6^), whereas CD63 (range 6.2 × 10^6^–2.9 × 10^7^) and CD41 (range 8.1 × 10^6^–4.1 × 10^7^) were in between the two. Analysis of the co‐localization of tetraspanins CD9, CD63 and CD81 on the CD41‐captured PEVs (Figure S6a) showed significant differences between the activation modes regarding the co‐localization of CD9 and CD9/CD63 with CD41 (Figure S6b). Compared to the US PEVs, expression of CD9 in the CD41‐positive PEVs was downregulated in the GPVI, CLEC‐2 and TC PEVs. In contrast, the GPVI and CLEC‐2 PEVs displayed upregulation of CD9/CD63 double positive PEVs compared to the US PEVs. The same trend was present in the TC PEVs, but the difference was not statistically significant (2‐way ANOVA followed by Tukey's multiple comparisons test). In all PEV types, the co‐expression of CD81 with CD41 was negligible.

To summarize, the basic EV particle characterization only revealed differences in particle yields, most notably between GPVI and CLEC‐2. GPVI was as potent as TC, whereas CLEC‐2 only weakly induced PEV formation compared to US PEVs. No differences in size distribution or morphology were observed. However, the tetraspanin co‐localization profiles among all the agonist‐induced PEV types were the same (e.g., externalization of CD63), and distinct from the profile of the US PEVs.

### 3.3 Profiling the PEV types through protein and miRNA omics analyses reveals only subtle, mostly quantitative differences

Next, we wanted to profile the underlying molecular fingerprints of the PEVs to explain the observed functional differences in the macrophages. To investigate the extent of the platelet's ability to tune the molecular cargo of PEVs receptor dependently, we performed three complementary analyses: protein mass spectrometry, PEA of proinflammatory proteins and miRNA sequencing.

**FIGURE 3:**
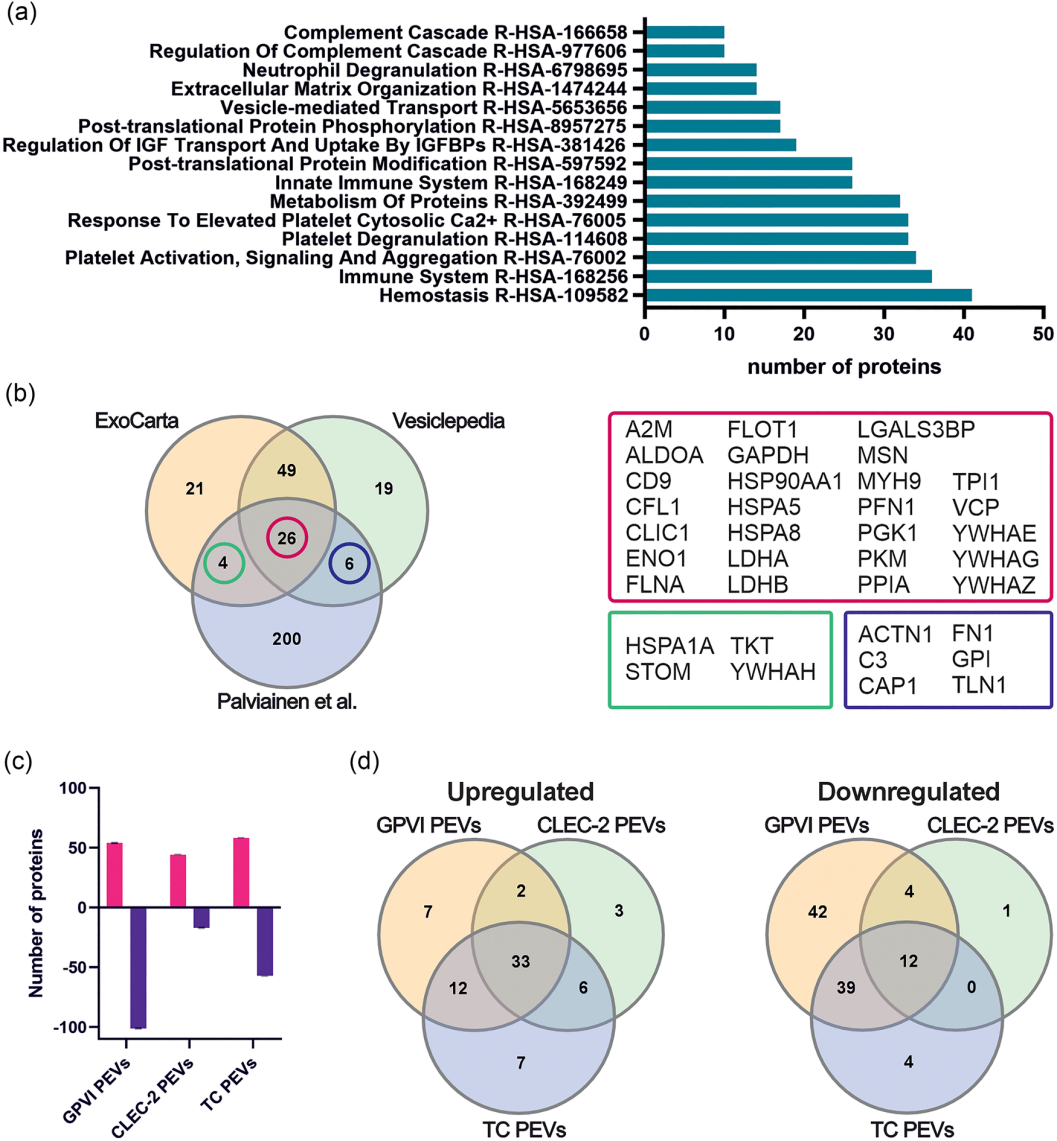
Comparison of the receptor‐induced differences in the protein cargo of PEVs analyzed with mass spectrometry (*n* = 3; biological replicates representing 12 donors). (a) Bar graph showing the top 15 pathways based on the number of proteins involved in the pathway (≥10, adjusted *p*‐value ≤ 0.05). (b) Venn diagram comparing the 240 identified proteins, ExoCarta top 100 and Vesiclepedia top 100 proteins reveals 26 proteins shared by the three datasets. In addition, this dataset shares four proteins with the ExoCarta top 100 proteins, and six with the Vesiclepedia top 100 proteins, respectively. (c) Bar graph illustrating the upregulated (red) and downregulated (purple) proteins when the proteomes of the GPVI, CLEC‐2 and TC PEVs were compared to the proteome of the US PEVs. Statistical significance was determined with multiple unpaired *t*‐tests with Benjamini, Krieger and Yekutieli test correction. (d) Venn diagrams of upregulated and downregulated proteins in the GPVI, CLEC‐2 and TC PEVs in comparison to the US PEVs. PEVs, platelet‐derived extracellular vesicles.

Firstly, the protein content of the PEVs was analyzed with mass spectrometry. In total, 250 proteins were identified from the samples (Table S3). Out of 250 proteins, 236 were converted to gene ID for further examination. A Reactome pathway analysis (Gillespie et al., 2022) of all proteins showed that, in addition to the expected platelet‐related pathways (e.g. R‐HSA‐114608, R‐HAS‐76005, R‐HAS‐76002), several immune system‐related pathways (e.g. complement cascade R‐HAS‐166658, regulation of complement cascade R‐HAS‐977606, and innate immune system R‐HAS‐168249 pathways) were in the top 15 enriched pathways (Figure 3a). Gene ontology analysis revealed that several proteins (YWHAE, VCP, C4BPA, ENO1, PARK7, CORO1A, HSP90B1, TUBA1B, TMSB4X, FLNA, YWHAG, PDIA3, HSPA8, HSP90AA1, ACTN1, MSN, NAP1L1, YWHAZ, PDIA4, PKM, ZYX, MYH9, CALR, P4HB, PFN1, ALDOA, PPIB, PPIA and HSPA1A) were associated with the protein corona and RNA binding (GO:0003723). Comparison of the proteome data to the top 100 protein lists of ExoCarta (Keerthikumar et al., 2016) and Vesiclepedia (Kalra et al., 2012) identified 36 proteins shared with either one or both of the databases (Figure 3b). These 36 proteins are known EV membrane proteins (e.g., CD9, CFL1 and FLOT1), or cytosolic proteins (e.g., ALDOA and GAPDH) indicating an enrichment of typical EV proteins in the PEVs (Théry et al., 2018). Next, the protein abundances of the GPVI, CLEC‐2 and TC PEVs were compared to the protein abundance of the US PEVs. A bar graph (Figure 3C) shows the differentially regulated proteins (Table S5) in the GPVI, CLEC‐2, and TC PEVs in relation to the proteins in the US PEVs. In the GPVI PEVs, 54 proteins were upregulated and 101 downregulated when compared to the US PEVs. The corresponding numbers were 44 and 17, and 57 and 58 for the CLEC‐2 and TC PEVs, respectively. The number of downregulated and upregulated proteins in the GPVI, CLEC‐2 and TC PEVs compared to the US PEVs are illustrated in Venn diagrams (Figure 3d), showing only minor differences between the proteomes of the receptor‐induced PEV types.

A large proportion of the proteins identified in this study have previously been reported as part of the molecular corona of EVs (Palviainen et al., 2020; Tóth et al., 2021), although the PEVs were induced from SEC‐isolated platelets. To further investigate the corona proteins of the PEVs, the current proteome was compared to the proteomes from two previous PEV proteome studies (Aatonen et al., 2014; Tóth et al., 2021). A Venn diagram shows the overlap in the identified proteins between the studies (Figure S7), and variations in methodologies are listed in Table S5. Gene ontology (GO) enrichment analysis was performed for the shared proteins in all datasets, or between any two datasets. Interestingly, 78 proteins were shared between all three studies despite the methodological differences. The molecular function of GO enrichment analysis revealed that most of the protein functions were related to binding of biomolecules (Figure S7), 48 proteins were shared between our study and Tóth et al. (Tóth et al., 2021) and 56 proteins between the studies by Aatonen et al. (Aatonen et al., 2014) and Tóth et al. These 104 proteins represent GO classes of functions for biomolecule binding and activity. Finally, shared proteomes between our study and that of Aatonen et al., where platelets had also been activated with an agonist (Aatonen et al., 2014), showed an overlap of 27 proteins with cytokine functions and neurotrophin binding.

**FIGURE 4:**
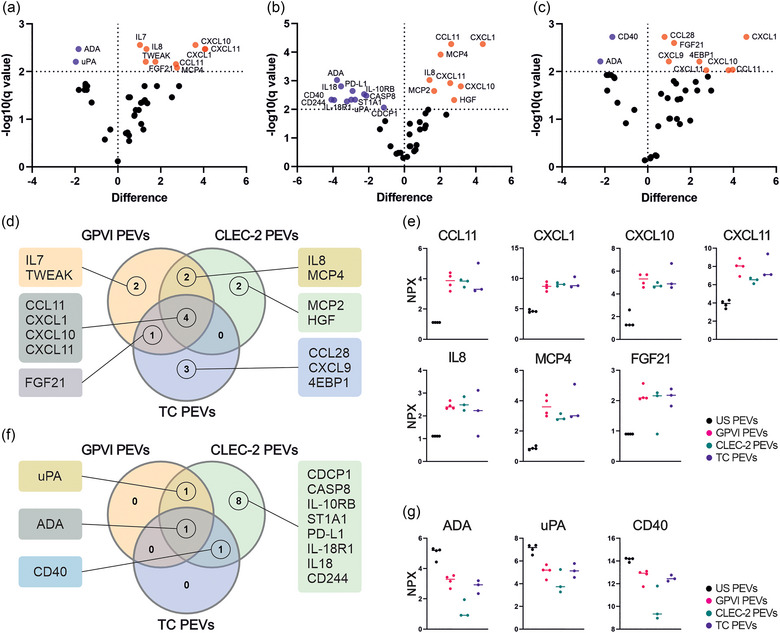
Quantitative analysis of the targets of the inflammation panel by PEA revealed distinctive receptor‐dependent tuning of the PEV proteins. PEVs isolated by iodixanol cushion ultracentrifugation were analysed for 80 inflammation panel targets. The GPVI (*n* = 4; biological replicates representing 16 donors), CLEC‐2 (*n* = 3; biological replicates representing 12 donors) and TC PEVs (*n* = 3; biological replicates representing 12 donors) were compared to the PEVs from unstimulated platelets (US PEVs, *n* = 4; biological replicates representing 16 donors). Statistical significance was determined with multiple unpaired *t*‐tests with Benjamini, Krieger and Yekutieli test correction. Results are presented as volcano plots for each PEV type displaying the differentially expressed inflammatory proteins compared with those in the US PEVs. Proteins are graphed by difference in means (SO 0.1, *x* axis) and significance (FDR *q* < 0.05, *y* axis). Proteins in orange are upregulated, and in purple downregulated compared to the proteins in the US PEVs. (a) Volcano plot of the GPVI PEVs compared to the US PEVs shows nine upregulated and two downregulated proteins. (b) Volcano plot of the CLEC‐2 PEVs compared to the US PEVs shows eight upregulated and 11 downregulated proteins. (c) Volcano plot of the TC PEVs compared to the US PEVs shows eight upregulated and two downregulated proteins. (d) Venn diagram of significantly upregulated proteins in the GPVI, CLEC‐2 and TC PEVs. (e) Plots illustrating the median expression of seven inflammatory proteins, CCL11, CXCL1. CXCL10, CXCL11, IL8 (CXCL8), MCP4 (CCL13), FGF21, which were upregulated in at least two PEV types. (f) Venn diagram of significantly downregulated proteins in the GPVI, CLEC‐2 and TC PEVs compared to the US PEVs. (g) Median expression of three inflammatory proteins, ADA, uPA and CD40, which were downregulated in at least two PEV types. Statistical significance was calculated with multiple unpaired *t*‐tests with Benjamini, Krieger and Yekutieli test correction to control the FDR. PEA, proximity extension assay; PEVs, platelet‐derived extracellular vesicles.

The functional assays in vivo and in vitro implicated the capacity of the PEVs to impact inflammation‐related events. Since the proteomic data also showed upregulation of immunity and inflammation‐related proteins, we further analyzed PEV proteins with PEA inflammation panels. PEA allowed quantitative comparisons of 80 proteins between the four PEV types (De Paoli et al., 2018). After exclusion of NPX values 50% below the assay's LOD, 41 proteins remained for quantitative comparison (Table S6). Volcano plots show the differentially expressed inflammation proteins in the GPVI PEVs (Figure 4a), CLEC‐2 PEVs (Figure 4b), and TC PEVs (Figure 4c) most clearly in relation to the US PEVs. In the GPVI PEVs, nine proteins were upregulated, and two proteins downregulated in comparison to the US PEVs. In the CLEC‐2 PEVs, eight proteins were upregulated and 11 downregulated, whereas in the TC PEVs eight proteins were upregulated and two downregulated in comparison to the US PEVs. A Venn diagram of the upregulated proteins (Figure 4d) shows seven proteins shared by at least two PEV types: CCL11, CXCL1, CXCL10, CXCL11, IL8 (CXCL‐8), MCP4 (CCL13), and fibroblast growth factor 21 (FGF21) (Figure 4e). A Venn diagram of the downregulated (Figure 4f) proteins reveals that adenosine deaminase (ADA), urokinase‐type plasminogen activator (uPA) and CD40 (Figure 4g) were shared between at least two PEV types, whereas up‐ or downregulated proteins that were present only in the GPVI, CLEC‐2 or TC PEVs are shown in Figure S8. Comparison of the PEVs from the ITAM‐receptor activated platelets revealed only quantitative differences in the cytokines, which were mostly downregulated in the CLEC‐2 PEVs versus the GPVI PEVs, which in turn exhibited similar cytokine profiles as the TC PEVs.

**FIGURE 5:**
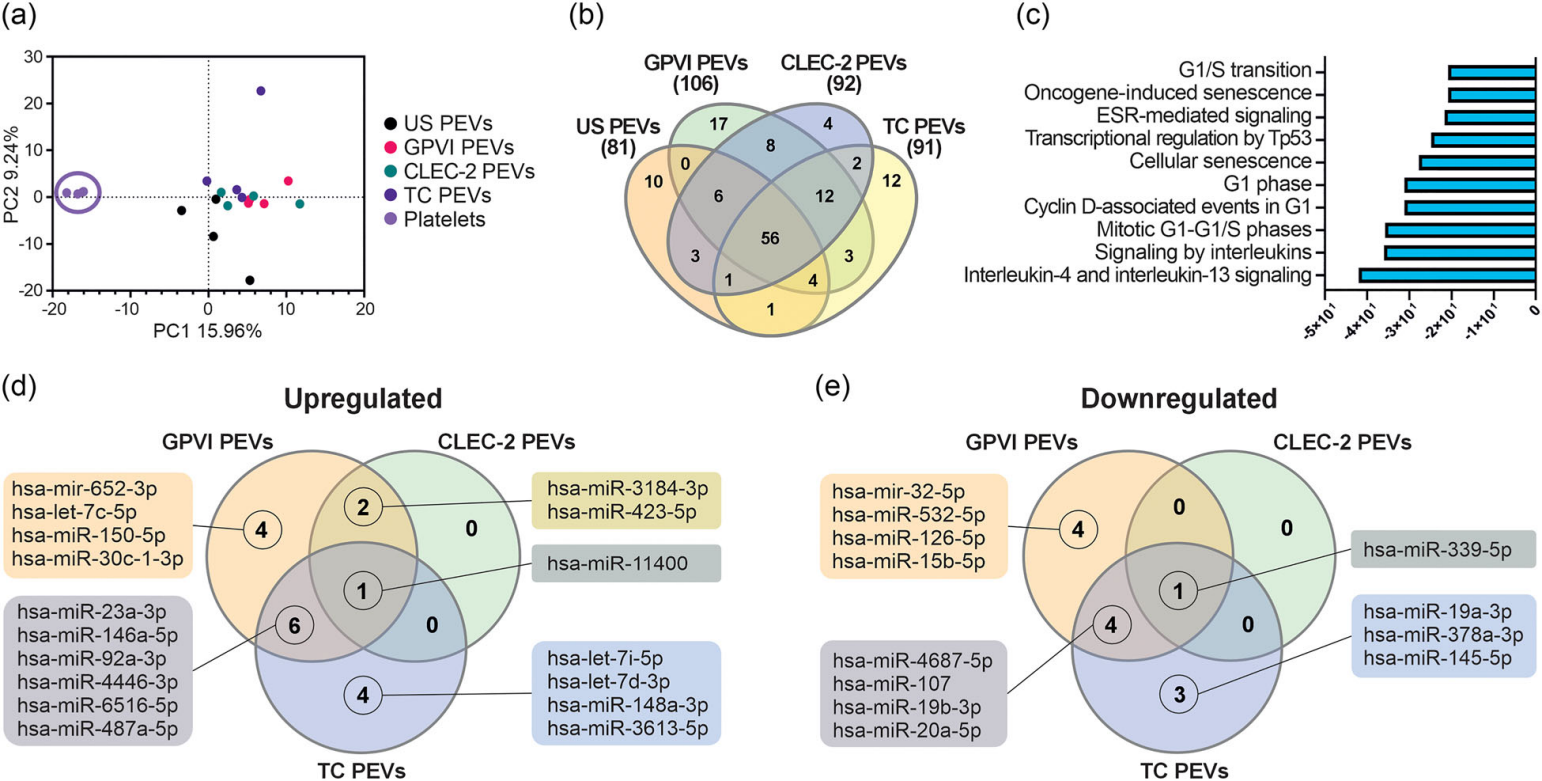
Comparison of the miRNA content of unstimulated platelets and the differentially induced PEVs (*n* = 4; biological replicates representing 16 donors). (a) Principal component analysis of the 541 shared miRNAs show quantitative differences between platelets and PEVs. The US, GPVI, CLEC‐2 and TC PEV miRNAs overlap with each other, and separation is only observed in comparison to the miRNAs of unstimulated platelets. (b) Venn diagram of the DEGs in the US, GPVI, CLEC‐2 and TC PEV miRNAs compared to the platelet miRNAs. Numbers of the differentially up‐ or downregulated miRNAs in PEVs in comparison to the miRNAs of unstimulated platelets are indicated in brackets. (c) Bar graph of the top 10 pathways (*p* ≤ 0.01) affected by the upregulated miRNAs in the differentially induced PEVs compared to platelets. Analysis was performed using the DESeq2 algorithm and the *p‐*values were corrected for multiple testing with FDR method. (d) Venn diagram of the upregulated miRNA DEGs of the GPVI, CLEC‐2 and TC PEVs compared to the US PEVs. (e) Venn diagram of the downregulated miRNA DEGs of the GPVI, CLEC‐2 and TC PEVs compared to the US PEVs. DEGs, differentially expressed genes; FDR, false discovery rate; PEVs, platelet‐derived extracellular vesicles.

Due to the richness of RNA‐binding proteins in the PEVs, we finally analysed the miRNA content of the PEVs as a possible mechanism of action for their different functionalities. miRNAs from unstimulated platelets and the four PEV types were sequenced, and 541 miRNAs were expressed across all the samples revealing no qualitative differences (Table S7). PCA analysis revealed separation in the miRNA expression profiles that differentiated the unstimulated platelets and PEVs (Figure 5a). Instead, contrary to our hypothesis, we found no separation among the miRNAs from the US, GPVI, CLEC‐2 or TC PEVs. We then analyzed the up‐ or downregulated miRNA differentially expressed genes (DEGs) of each PEV type in comparison to platelet miRNAs using the DESeq2 algorithm (Love et al., 2014). While a total of 56 DEGs were shared among all four PEV types when compared to platelets, unique DEGs were distributed as follows: 10 in US PEVs, 17 in GPVI PEVs, 4 in CLEC‐2 PEVs, and 12 in TC PEVs (Figure 5b). Next, these DEGs were analyzed based on the pathways affected by their target genes. The top 10 pathways affected by the shared DEGs from all the PEV types were interleukin‐4 and interleukin‐13 signaling (R‐HSA‐6785807), signaling by interleukins (R‐HSA‐449147), mitotic G1‐G1/S phases (R‐HSA‐453279), cyclin D‐associated events in G1 (R‐HSA‐69231), G1 phase (R‐HSA‐69236), cellular senescence (R‐HSA‐2559583), transcriptional regulation by Tp53 (R‐HSA‐3700989), ESR‐mediated signaling (R‐HSA‐8939211), oncogene‐induced senescence (R‐HSA‐2559585), and G1/S transition (R‐HSA‐69206) (Figure 5c). Finally, we compared the GPVI, CLEC‐2 and TC PEV miRNAs by analysing their up‐ and downregulation against the US PEV miRNAs. This analysis revealed only minor quantitative differences: the CLEC‐2 PEVs had three differentially upregulated and one downregulated miRNA when compared to the US PEVs, the GPVI PEVs had 13 up‐ and nine downregulated, and the TC PEVs had 11 up‐ and eight downregulated miRNAs and few of them were unique for the GPVI, CLEC‐2 or TC PEVs (Figure 5d,e). This data indicates that the PEV miRNAs may impact many functions including immunomodulatory ones, but the miRNA signatures did not allow identification of specific profiles associated with the PEV‐generating signal in this study.

As a summary of the protein and miRNA content analyses, we found that the PEVs were tunable by platelet activation, and that the US PEVs exhibited their own distinct molecular profile. However, most of the differences between the PEVs from the receptor activated platelets were quantitative, for example, differences in the cytokine concentrations. The few unique qualitative differences did not allow prediction of a certain functional profile for the different PEV types.

## 4 DISCUSSION

Although platelets can be induced to generate EVs through differential receptor mediated activation, little is yet known about their immunomodulatory capacity. In vitro studies have highlighted monocytes and macrophages as targets of particular interest for PEV‐mediated immunomodulation (Ilvonen et al., 2024; Laffont et al., 2016; Sadallah et al., 2011; Song et al., 2022; Tersteeg et al., 2014). However, it is technically challenging in vivo to visualize the dynamic interaction between PEVs and cells at high spatiotemporal resolution. Therefore, we used a recently established zebrafish embryo model of the vertebrate reticuloendothelial system (Campbell et al., 2018; Hayashi et al., 2020; Pattipeiluhu et al., 2022) to study interactions of IV injected PEVs with cells accessible via circulation. This, to our knowledge, is the first study where human PEVs were injected into zebrafish embryos. The in vivo imaging showed that PEVs were clearly more accumulated by macrophages than by other cells, in particular scavenger endothelial cells. We find this interesting since it has previously been reported that the IV injected EVs and synthetic nanoparticles are preferentially accumulated in scavenger endothelial cells in zebrafish embryos. Indeed, a diverse array of nanoparticles screened by us (Hayashi et al., 2020) and others (Campbell et al., 2018) were almost always primarily sequestered by these cells, whereas the contribution of macrophages was minor (<20% of the overall sequestration of IV injected 70 nm SiO_2_ nanoparticles) (Hayashi et al., 2020). The dominant role of scavenger endothelial cells in nanoparticle sequestration in zebrafish embryos has been reciprocated in rodent models where liver sinusoidal endothelial cells, the mammalian functional equivalent of zebrafish scavenger endothelial cells, may contribute more than Kupffer cells (macrophages) to the hepatic clearance of systemically administered nanoparticles (Campbell et al., 2018; Hayashi et al., 2020; Pattipeiluhu et al., 2022). This seems to also apply to some EVs, as zebrafish melanoma EVs (IV injected) (Hyenne et al., 2019) and yolk syncytial layer EVs (secreted in vivo) (Verweij et al., 2019) had the same homing pattern as nanoparticles. This makes our finding of PEV homing distinct from previous reports on EVs of different origin, implying that not all EV types behave the same way in circulation. We think that two mechanisms may play a role here: preferential uptake of PEVs by macrophages as previously reported using mammalian cell models in vitro (Laffont et al., 2016; Sadallah et al., 2011; Song et al., 2022), *ex vivo* (Ilvonen et al., 2024), and in vivo (Li et al., 2022), or possibly faster degradation of PEVs in endolysosomes due to rapid acidification of scavenger endothelial cells as previously observed for nanoparticles with a preformed, fluorescently labeled protein corona (with a maximal pH decrease at 5 mpi) (Mohammad‐Beigi et al., 2020). Furthermore, the rapidity of PEV sequestration from the bloodstream shown at 3 mpi onwards (Figure 1e) implies that macrophages may outcompete scavenger endothelial cells in PEV uptake when the time frame and dose are limited, whereas with higher PEV numbers, scavenger endothelial cells could serve as a greater EV sink from circulation. Sequestration kinetics of PEVs and other cells await further in vivo studies together with determination of the ligand‐receptor pairs involved in the uptake. However, here we clearly demonstrated that PEVs home in on macrophages as the primary target in vivo supporting previous human cell data (Ilvonen et al., 2024; Laffont et al., 2016; Sadallah et al., 2011; Song et al., 2022).

One limitation of the zebrafish model is the greater evolutionary distance from humans compared to mammalian rodent models, which may even be humanized to express cognate receptors. However, many receptors involved in thrombosis and innate immune cell interactions are actually highly conserved between zebrafish and humans, as exemplified by the capability of thrombocytes, the functional equivalents of human platelets, to express the primary platelet surface receptor CD41 (*itga2b*) and to mediate clotting (Khandekar et al., 2012; Lin et al., 2005). Furthermore, most of the PEV surface receptors implicated in human platelet‐monocyte interactions (CD42b, CD61 and P‐selectin) have zebrafish orthologs, and their interaction partners (CD11b/CD18 and PSGL‐1; *itgam/itgb2* and *selplg* in zebrafish) are also expressed in the macrophages of 2–3 dpf zebrafish embryos (Lange et al., 2023). Similarly, they express members of the TIM and TAM receptor families (Lange et al., 2023) that, in humans, are known to recognize phosphatidylserine on PEVs (Naeini et al., 2020). Although the precise molecular determinants of the interaction between human PEVs and zebrafish macrophages were not explored in this study, the discovery of macrophages exhibiting preferential PEV uptake and their subsequent polarization into a *tnfa*‐expressing phenotype offers an intriguing insight into a conserved machinery that recognizes homologous molecular patterns, paving the way for future PEV studies with the zebrafish model.

The functional in vitro macrophage assay showed that the four PEV types elicited distinct secretomes. This highlights the significant impact of subtle molecular tuning within seemingly similar PEVs. Notably, neither conventional EV characterization following established guidelines (e.g., MISEV (Théry et al., 2018)), nor omics analyses of proteins and miRNA yielded distinct profiles that could predict the mechanisms of action of the different PEVs. The original objective of this study was to compare two ITAM receptors in platelets, GPVI and CLEC‐2 (Rayes et al., 2019) sharing downstream signaling pathways, which are also analogous for the immune receptor signaling in myeloid, plasma dendritic, B‐ and T‐cells. Importantly, the engagement of GPVI in circulation takes place under completely different pathophysiological conditions than that of CLEC‐2. According to our hypothesis, we expected to discover distinct PEVomes upon stimulation of platelets via GPVI and CLEC‐2 if platelets were to engage in immunoregulation. Indeed, this was the case with the marked functional differences in macrophage secretomes and the differences in PEV yield, as GPVI induced more PEVs compared to CLEC‐2 (unsurprisingly, stimulation through any of the receptors increased the PEV yield compared to unstimulated platelets). In contrast, size distributions of the PEVs were invariant with predominantly smaller PEVs (<100 nm), as per the power law distribution of EVs (Arab et al., 2021; Paulaitis et al., 2018). However, with the used methods (NTA, MRPS or SP‐IRIS), we cannot rule out that differences in PEVs < 50 nm could be detected with methods such as asymmetric flow field‐flow fractionation (Multia et al., 2019). Surprisingly, the molecular cargo differences between the GPVI and CLEC‐2 PEVs were minor. Despite a trend of lower cytokine levels in the CLEC‐2 PEVs by PEA (Figures 4f,g and S7d), there were no statistically significant differences between these two PEV types and only one miRNA (hsa‐miR‐140‐5p) was significantly upregulated in the GPVI PEVs compared to the CLEC‐2 PEVs. There are a few explanations for the minor differences between the PEV types from activated platelets. Firstly, only protein and miRNA analyses were conducted in this study, and it is possible that significant differences could exist in the lipidome, metabolome or glycosignatures of these PEVs. Secondly, the number of identifications, especially in the proteomic data, may explain the low number of differentially expressed molecules. Moreover, the more abundant proteins or miRNAs may underlie ‘common functions,’ whereas any ‘regulatory’ molecules executing the different functional profiles could remain undetected due to lower levels of expression. It is also possible that the secondary activation from engagement of CD41/61 or autocrine signaling (e.g., ADP, TXA2) could mask initiating receptor‐specific molecular features, which warrants future studies with receptor antagonists and signaling inhibitors. Thirdly, future integrative multiomics analyses may reveal more complex, molecular signatures, especially when performed on single EVs or EV subpopulations. In this study, we deliberately chose to include all PEV subpopulations as a batch for studying immunomodulatory effects holistically. Finally, we believe that in vivo PEV functionality likely involves engagement of multiple receptors and signaling pathways by different biomolecule types and alterations in their quantities, making the stimulatory processes even more complicated. In any case, our data underscores two pivotal concepts (i) platelets actively regulate immune cell coordination by tuning the PEVomes, and (ii) the analysis of the molecular content and physicochemical characteristics of PEVs alone inadequately defines their function. Thus, these results highlight the multifaceted role of EVs as complex messengers and challenge the notion that analysis confined to single omics or basic EV characteristics is sufficient to reveal the mechanism of action or predict function. Therefore, we advocate for a broader perspective in molecular analyses and the need for functional assays.

Interestingly, the US PEVs formed *in the absence of* an exogenous activator induced a unique macrophage secretome. Furthermore, the US PEVs had significantly distinct molecular content compared to the PEVs from activated platelets as shown by proteomic analyses (Figure 3c,d, Figure 4). The US PEVs exhibited higher levels of 12 proteins (compared to the GPVI, CLEC‐2 or TC PEVs), nine of which are known constituents of the biomolecular corona of circulating EVs (Palviainen et al., 2020; Tóth et al., 2021) (A2M, APOA1, APOA4, APOB, APOE, HBB, HPX, ORM1, SERPINA3), and the remaining three are membrane proteins in platelets (CD36, FLOT1, FLOT2). This suggests that the US PEVs originate from the platelet plasma membrane, which is further supported by the loss of 101 proteins in the TC and GPVI‐induced PEVs when compared to the US PEVs including immunoglobulins, complement components, and heat shock proteins, all known constituents of the biomolecular corona and of plasma membrane origin. The plasma membrane origin of the US PEVs is also supported by the tetraspanin co‐localization profiles: in comparison to the US PEVs, receptor‐mediated activation of platelets decreased the percentage of CD41+/CD9+ PEVs and increased the percentage of triple positive CD41+/CD9+/CD63+ PEVs. Our finding is corroborated by a previous study showing an increase in double positive CD41+/CD9+ EVs in plasma and triple positive CD41+/CD9+/CD63+ EVs in serum (Karimi et al., 2022) (coagulation activates platelets generating more platelet EVs in serum than in plasma [George et al., 1982; Palviainen et al., 2020]). Although we cannot fully exclude inadvertent stimulation by, for example, autocrine ADP release during platelet handling, our data did not show platelet activation and the activation time to induce PEVs was purposely kept short (30 min) to reduce the possibility of PEVs being generated by aging, apoptosis, or necrosis. In support of a notion of a constitutive release of PEVs, previous studies have shown liberation of radioactively labeled platelet membrane in rabbit circulation (George et al., 1976) and PEV generation over time in vitro (Aatonen et al., 2014; Cauwenberghs et al., 2006; Tóth et al., 2021) and *ex vivo* in platelet concentrates over the course of several days (Böing et al., 2008; Valkonen et al., 2019). Therefore, we put forward, that platelets, like other cells, also constitutively release small amounts of EVs into the extracellular milieu. Such PEVs could have a homeostatic role in a paracrine manner, and therefore not be readily detected in circulation. Homeostatic formation of EVs is a newly emerging concept in the EV field (Stratman et al., 2022; Zierden et al., 2023).

Within the last years the knowledge of EV heterogeneity and the existence of various subpopulations with variable biogenetic origins has grown explosively. However, there are still very few systematic comparisons of the effect of different activation routes on the EVome from the same cell type. Platelets represent an excellent cell model for studying the impact of different signaling pathways on the EVome. Therefore, exploration of the tunability of the PEVome in the context of the immune system is relevant for the possibility to tune the PEVs towards a defined profile, which would be of interest for the development of drug delivery systems or advanced molecular therapeutic products (AMTPs) with innate therapeutic capacity (Burnouf et al., 2023). Also, the high interest in the diagnostic biomarker use of PEVs still demands a better understanding of the variability of the PEVome and its relation to the PEV‐inducing signal, whether it is, for example, viral by CLEC‐2 (Garcia et al., 2022), thrombotic, mediated by thrombin and collagen receptors (Maguire et al., 2020), or singularly by GPVI. Our findings indicate that tuned PEVs are released in vitro both through differential receptor activation and constitutively in the absence of external stimulation. Understanding their functional immunomodulatory roles and mechanism of action requires more than just comparative physicochemical characterizations or omics approaches. Instead, we propose that developing sensitive functional assays can unveil the immunomodulatory roles executed by even subtle distinctions among PEVomes.

## AUTHOR CONTRIBUTIONS

**Mari Palviainen**: Conceptualization; data curation; formal analysis; funding acquisition; investigation; methodology; project administration; supervision; visualization; writing—original draft; writing—review and editing. **Johanna Puutio**: Formal analysis; investigation; project administration; validation; visualization; writing—original draft; writing—review and editing. **Rikke Halse Østergaard**: Formal analysis; investigation; visualization; writing—original draft; writing—review and editing. **Johannes A. Eble**: Resources; writing—review and editing. **Katariina Maaninka**: Investigation; validation; writing—review and editing. **Umar Butt**: Formal analysis; investigation; visualization; writing—review and editing. **Joseph Ndika**: Investigation; methodology. **Otto K. Kari**: Methodology. **Masood Kamali‐Moghaddam**: Investigation; writing—review and editing. **Kasper Kjaer‐Sorensen**: Resources. **Claus Oxvig**: Resources. **Ana M. Aransay**: Formal analysis; investigation; resources. **Juan M. Falcon‐Perez**: Resources. **Antonio Federico**: Data curation; formal analysis; writing—review and editing. **Dario Greco**: Formal analysis; resources; writing—review and editing. **Saara Laitinen**: Resources; writing—review and editing. **Yuya Hayashi**: Data curation; formal analysis; investigation; methodology; supervision; visualization; writing—original draft; writing—review and editing. **Pia R**.‐**M Siljander**: Conceptualization; funding acquisition; project administration; supervision; writing—original draft; writing—review and editing.

## CONFLICT OF INTEREST STATEMENT

The authors declare no conflicts of interest.

## Supporting information



Supporting Information

## Data Availability

The mass spectrometry proteomics data have been deposited to the ProteomeXchange Consortium via the PRIDE partner repository with the dataset identifier PXD047323. The RNA‐Seq data have been deposited to the GEO NCBI repository with the accession number GSE184577.
