# Correction to “Beyond basic characterization and omics: Immunomodulatory roles of platelet‐derived extracellular vesicles unveiled by functional testing”

**DOI:** 10.1002/jev2.70015

**Published:** 2024-12-13

Palviainen, M., Puutio, J., Østergaard, R. H., Eble, J. A., Maaninka, K., Butt, U., Ndika, J., Kari, O. K., Kamali‐Moghaddam, M., Kjaer‐Sorensen, K., Oxvig, C., Aransay, A. M., Falcon‐Perez, J. M., Federico, A., Greco, D., Laitinen, S., Hayashi, Y., & Siljander, P. R.‐M. (2024). Beyond basic characterization and omics: Immunomodulatory roles of platelet‐derived extracellular vesicles unveiled by functional testing. *Journal of Extracellular Vesicles*, *13*, e12513. https://doi.org/10.1002/jev2.12513


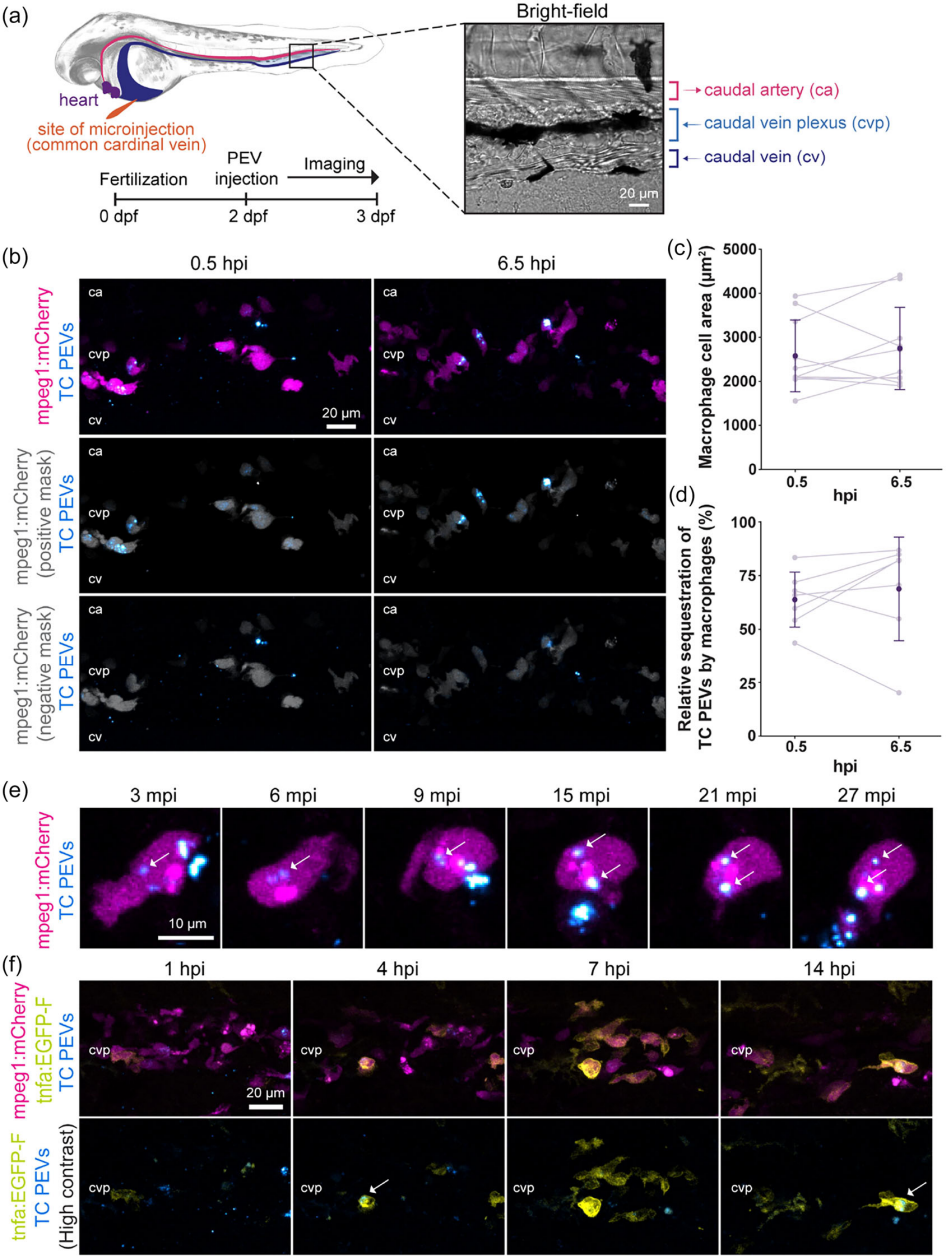


In Figure 1(f), the 14 hpi column showed the same image in the first row and the second row, whereas it should instead show a high contrast image of the first row following a different colour scheme as correctly shown in the other three columns (1, 4 and 7 hpi). This has been corrected by replacing the image in question. This does not affect the scientific content or the conclusion, since the results have already been fully presented in the first row of images, whereas the second row shows the same results with visual enhancement to highlight two of the three colours for clarity. There was also a typo in the label for the second row “(High constrast)”, which should have read “(High contrast)”.

In Figure 1(a), in the bright‐field image, the scale bar was not fully shown. This has also been corrected by adjusting its position.

We apologize for these errors.

