# Supplementary material for: Beyond basic characterization and omics: Immunomodulatory roles of platelet‐derived extracellular vesicles unveiled by functional testing
Source: J Extracell Vesicles. 2024 Sep 27;13(10):e12513. doi: 10.1002/jev2.12513 (PMC11428872; doi:10.1002/jev2.12513)
Supplement: Supplementary file 1 — Supporting Information [file JEV2-13-e12513-s001.zip › jev212513-sup-0001-SuppMat.docx]

# Supplementary material

# Beyond Basic Characterization and Omics: Immunomodulatory Roles of Platelet-Derived Extracellular Vesicles Unveiled by Functional Testing ­­­

**Supplementary Figure legends**

**Figure S1.** Workflow for platelet isolation, activation, and isolation of PEVs. Platelets were isolated by gel filtration from fresh leukocyte-reduced platelet concentrates derived from the buffy coats of four ABO RhD-matched blood donors. Isolated platelets were incubated 30 min at 37 °C with CRP-XL (CRP), rhodocytin (Rh), and thrombin and collagen co-stimulus (TC), or without any exogenous agonist to produce GPVI, CLEC-2, TC and unstimulated (US) PEVs, respectively. After careful platelet removal, PEVs were isolated with iodixanol cushion ultracentrifugation, resuspended and concentrated with ultrafiltration. Figure created with BioRender.com. PEVs, platelet-derived extracellular vesicles.

**Figure S2.** SEM and high sensitivity FC analysis of platelets and PEVs. **A.** SEM micrographs of unstimulated platelets and platelets activated via GPVI, CLEC-2, and TC co-stimulation. Micrographs show typical morphological changes of activated platelets and PEV shedding. Scale bars = 5 µm. **B.** Gating strategy for PEVs, CRs, and platelets in FC analysis. Representative scatter plots a.–e. show gating of **a.** PEV (200–1000 nm), CR (1000–2,000 nm) and PLT (2000–3000 nm) populations based on particle diameter derived from light scattering from unstained sample, **b.** CD62P+ events determined by **c.** mouse IgG1-PE (isotype control) staining, **e.** events positive for PAC-1 binding to the active CD41/CD61 complex determined by **d.** mouse-IgM-FITC (isotype control) staining. Particle diameters were derived from light scatter data via calibration with the Rosetta calibration system (Exometry, The Netherlands). Fluorescence gates were set based on isotype controls to differentiate between specific and nonspecific binding of antibodies. Fluorescent events exceeding the gate were determined as positive (+) events. **C.** Analysis of platelet activation with FC. Platelets were activated for 30 min and the concentrations of total, CD62P+ and PAC-1+ events in the platelet gate (2000–3000 nm in diameter) was measured immediately (unlabeled sample, left panel) or after labeling with CD62P and PAC-1 antibodies for 2 h. The data is presented as the concentration of total platelets (left panel) and as percentages of CD62P+ and PAC-1+ platelets out of total platelets. Upon receptor-mediated activation, the total number of events in the platelet gate decreased significantly while the percentage of CD62P+ and PAC-1+ events increased compared to unstimulated platelets. Bar graphs represent means ± SD (n = 3; biological replicates representing 12 donors), ^*^*p* ≤ 0.05, ^**^*p* < 0.001, ^****^*p* < 0.0001 as determined by 2-way ANOVA followed by Tukey’s multiple comparisons test. **D.** FC analysis of the formation of GPVI, TC and CLEC-2 induced PEVs as a function of time. The concentration of CD61+ PEVs was measured at 15, 30, 60 and 180 min (left panel, representative image). The 30 min timepoint produced the highest PEV yield for CLEC-2 activation and was chosen for further experiments. The right panel shows the concentration of CD62P+ PEVs at 30 min (n = 3; biological replicates representing 12 donors, ^*^*p* ≤ 0.05). The CD61+ and CD62P+ PEVs represent events in the PEV gate (200–1000 nm in diameter) that exceed the fluorescence threshold. CRs, cell remnants; FC, flow cytometry; PEVs, platelet-derived extracellular vesicles; PLTs, platelets; SEM, scanning electron microscopy; TC, thrombin and collagen.

**Figure S3.** Interaction of macrophages with PEVs *in vivo*. Fluorescently labeled PEVs from platelets activated via GPVI stimulation were intravenously injected into zebrafish embryos at 2 dpf and live-imaged. **A.** *Tg(mpeg1:mCherry)* embryos were imaged every 20 min from 0.5 hpi to 6.5 hpi. Representative images show the total GPVI PEV signals (cyan) and macrophages (magenta) at two time points (top panel). The two lower panels display the same images after segmentation by a macrophage-specific mask (gray) to determine PEV colocalization with macrophage reporters in spatial *x-y-z* dimensions. The panel with the ‘positive mask’ shows only GPVI PEV signals colocalizing with macrophages while the ‘negative mask’ panel reveals those that are excluded, such as associations with cells other than macrophages. **B-D.** Image analysis results of TC *versus* GPVI PEVs. Thin and thick lines represent the individual data (n = 10 embryos) and mean values (n = 8 for GPVI PEVs and n = 7 for TC PEVs after excluding embryos where macrophages were moving in and out of the field of view), respectively. Total FI (fluorescence intensity) values show the relative difference of TC *versus* GPVI PEVs sequestered (**B**). Relative PEV sequestration by macrophages (**D**) is the area ratio of the macrophage-masked PEVs to the total PEVs. dpf, days post-fertilization; GPVI, glycoprotein VI; hpi, hours post-injection; mpi, minutes post-injection; PEVs, platelet-derived extracellular vesicles; TC, thrombin and collagen.

**Figure S4.** Uptake and localisation of PEVs in macrophages. **A.** Quantification of CellTrace^TM^ Far Red -labeled PEV signal in the cytoplasm (left panel) and on the cell membrane (right panel) of PMA-differentiated THP-1 cells (macrophages) at four time points. CSFE-labeled cells (0.5 x 10^5^ cells) were treated with 2.5 x 10^8^–5.0 x 10^8^ PEVs (n = 4; biological replicates representing 16 donors) and incubated for 3, 6, 9 or 12 h after which the cells were washed and fixed with 4% paraformaldehyde. Membranes were stained with anti-CD18 antibody. Imaging was performed with the PerkinElmer Opera Phenix High Content Screening System. Graphs represent percentages (mean $\pm$SEM) of THP-1 cells where cytoplasmic or membrane stain signal co-localized with the PEV-signal and at least 700 cells were counted per sample. The uptake of CLEC-2 PEVs into the cytoplasm was significant at 9 and 12 h compared to the mock treatment (^*^*p* ≤ 0.05). Statistical significance was calculated using two-way ANOVA with Bonferroni correction for multiple comparisons. **B.** Representative images of PEV colocalization with macrophages at 3 and 12 h. Mock treatment, PEVs from unstimulated platelets (US PEVs, 3 h) and CLEC-2 induced PEVs (CLEC-2 PEVs, 3 and 12 h) stained with CellTrace^TM^ Far Red (red) are shown with macrophages whose cytoplasms were stained with CellTrace^TM^ CFSE (green) and membranes with anti-CD18 antibody (yellow). Merged representative images of total cells with PEVs show PEVs in the cytoplasm and PEVs on the cell membrane (denoted with white arrows). Magnification 20X, scale bars = 1000 µm. Inserts show PEVs visible in cells and on the cell membrane, respectively (denoted with white arrows). **C.** Zoomed images of the same cell show presence of PEVs in the cytoplasm (white asterisk) and their simultaneous absence on the cell membrane and vice versa (white arrow). Scale bars = 50 µm. PEVs, platelet-derived extracellular vesicles.

**Figure S5.** Potency of platelet activation via different receptors in inducing the formation PEVs and analysis of size distributions. The morphology of the PEVs was compared with TEM and particle concentrations were measured with NTA and MRPS. Size distributions were studied with NTA, MRPS and SP-IRIS. **A.** TEM micrographs of negatively stained GPVI, CLEC-2, TC PEVs and PEVs released from unstimulated platelets display typical sizes and morphology for EVs. No differences were observed. **B.** Particle concentrations of PEVs from unstimulated platelets, and those from platelets activated through the GPVI receptor (GPVI PEVs), the CLEC-2 receptor (CLEC-2 PEVs), or via TC receptors (TC PEVs) were measured with NTA and MRPS. Bar graphs represent an average of all analyzed PEV isolates for NTA (n = 6; biological replicates representing 24 donors) and MPRS (n = 3; biological replicates representing 12 donors). PEV concentration is given as PEVs / 2.5 x 10^8^ platelets (average concentration in blood) used for the experiment. NTA showed a statistically significant increase in the formation of TC and GPVI PEVs, but not CLEC-2 PEVs, when compared to PEVs from unstimulated platelets (*p* ≤ 0.001; Kruskal-Wallis test followed by Dunn’s multiple comparison test). A similar trend was measured with MRPS, although no statistical significance was observed. **C.** Size distributions of the PEVs were measured with NTA, MRPS, and SP-IRIS. The recorded size distributions with NTA and MRPS showed similar size profiles for all PEVs. The data was sorted into 5 nm bins and analyzed with the Kruskal-Wallis test followed by Dunn’s multiple comparison test, but no significant differences were found (data not shown). Interferometry-based sizing (SP-IRIS) and counting of PEVs captured with anti-CD41 antibody also showed similar size distribution profiles for all PEVs (n = 3; biological replicates representing 12 donors). Binned data was analyzed as described above, and no statistical differences were observed (data not shown). Figure table: The mean and mode size of PEVs (nm ± SD) measured with NTA, MRPS and SP-IRIS. MRPS, microfluidic resistive pulse sensing; NTA, nanoparticle tracking analysis; PEVs, platelet-derived extracellular vesicles; SP-IRIS, single-particle interferometric reflectance imaging sensor; TC, thrombin and collagen; TEM, transmission electron microscopy.

**Figure S6.** Tetraspanin profiles of CD41-captured PEVs generated via activations of different platelet receptors or in the absence of an exogenous activator. The PEVs were analyzed with SP-IRIS using the ExoView R100 platform. **A.** Representative image of CD41-captured particles labeled with CD9, CD81 and CD63 antibodies. Source areas for the composite image of co-localized fluorescence markers is shown with a dotted line **B.** Percentages of CD41-positive PEVs labeled with CD9, CD63, and CD81 antibodies. Colocalization profiles of the CD41-captured, CD9-positive and CD41-captured, CD9/CD63-double positive PEVs showed statistically significant differences between the PEV types generated by activating platelets via the GPVI (GPVI PEVs), the CLEC-2 (CLEC-2 PEVs) and thrombin and collagen receptors (TC PEVs) compared to the PEVs from unstimulated platelets (US PEVs). Graphs represent means ± SD (n = 3; biological replicates representing 12 donors), ^*^*p* ≤ 0.05, ^**^*p* < 0.001, ^****^*p* < 0.0001 as determined by 2-way ANOVA followed by Tukey’s multiple comparisons test. PEVs, platelet-derived extracellular vesicles; SP-IRIS, single particle interferometric reflectance imaging sensor.

**Figure S7.** Proteomic data comparison of this study with two previous studies of PEVs by Aatonen et al. and Tóth et al. Venn diagram shows the overlap between the proteomes. Proteins shared by at least two studies were subjected to molecular function of GO enrichment analysis. Bar graphs represent top 10 molecular functions (*p* ≤ 0.01). GO, gene ontology; PEVs, platelet-derived extracellular vesicles.

**Figure S8.** Box plots of differentially expressed inflammation-related proteins in PEVs by PEA. **A.** Two proteins, IL7 and TWEAK, were significantly upregulated in the GPVI PEVs (n = 4; biological replicates representing 16 donors) compared to the US PEVs (n = 4; biological replicates representing 16 donors). **B.** Two proteins, MCP2 and HGF, were significantly upregulated in the CLEC-2 PEVs (n = 3; biological replicates representing 12 donors) compared to the US PEVs. **C.** Three proteins, CCL28, CXCL9 and 4EPB1, were significantly upregulated in the TC PEVs (n = 3; biological replicates representing 12 donors) compared to the US PEVs. **D.** Eight proteins, CDCP1, CASP8, IL-10RB, ST1A1, PD-L1, IL-18R1, IL18 and CD244, were significantly downregulated in the CLEC-2 PEVs compared to the US PEVs. Statistical significance was calculated with multiple unpaired *t*-tests with Benjamini, Krieger and Yekutieli test correction to control the FDR, and *p* ≤ 0.05 was considered significant. PEA, proximity extension assay; PEVs, platelet-derived extracellular vesicles; TC, thrombin and collagen; US, unstimulated.

**Supplementary Movies**

**Movie 1.** Platelet-derived extracellular vesicle (PEV)-macrophage interactions at 0.5 – 6.5 h post injection (hpi). *Tg(mpeg1:mCherry)* embryos were injected with the fluorescently labeled TC PEVs and imaged every 20 min starting at 0.5 hpi. Representative movies are shown. PEVs are seen in cyan and *mpeg1:mCherry* macrophages in magenta. Anterior left, dorsal top.

**Movie 2.** Platelet-derived extracellular vesicle (PEV)-macrophage interactions at 0.5 – 6.5 h post injection (hpi). *Tg(mpeg1:mCherry)* embryos were injected with the fluorescently labeled GPVI PEVs and imaged every 20 min starting at 0.5 hpi. Representative movies are shown. PEVs are seen in cyan and *mpeg1:mCherry* macrophages in magenta. Anterior left, dorsal top.

**Movie 3.** *tnfa*-induction in macrophages by platelet-derived extracellular vesicles (PEVs) at 1 – 14 h post injection (hpi). *Tg(mpeg1:mCherry);Tg(tnfa:EGFP-F)* embryos were injected with the fluorescently labeled TC PEVs and imaged every 30 min starting at 1 hpi. The movie shows sequestration of PEVs and *tnfa*-induction over time. TC PEVs are seen in cyan, *mpeg1:mCherry* in magenta and *tnfa:EGPF-F* in yellow. Anterior left, dorsal top.

**Supplementary Tables**

**Table S1.** List of proteins targeted in the proximity extension assay (inflammation panel).

**Table S2.** Macrophage secretomes (cytokines and chemokines) after 6 and 24 h of PEV treatment analyzed by a multiplex immunoassay.

**Table S3.** Table of the 250 identified proteins from proteomics analysis of PEVs.

**Table S4.** Comparison of identified proteins from proteomics analysis of the GPVI, CLEC-2 and TC PEVs.

**Table S5.** Comparison of platelet and platelet-derived extracellular vesicle methods used in the studies by Aatonen et al., Tóth et al., and the present study.

**Table S6.** List of proteins identified from the different PEVs by the proximity extension assay (inflammation panel). Proteins with NPX values < 50% above the limit of detection were included in the analysis.

**Table S7.** Table of the 541 identified platelet and platelet-derived extracellular vesicle miRNAs.
