# Supplementary figures and images for: Beyond basic characterization and omics: Immunomodulatory roles of platelet‐derived extracellular vesicles unveiled by functional testing
Source: J Extracell Vesicles. 2024 Sep 27;13(10):e12513. doi: 10.1002/jev2.12513 (PMC11428872; doi:10.1002/jev2.12513)

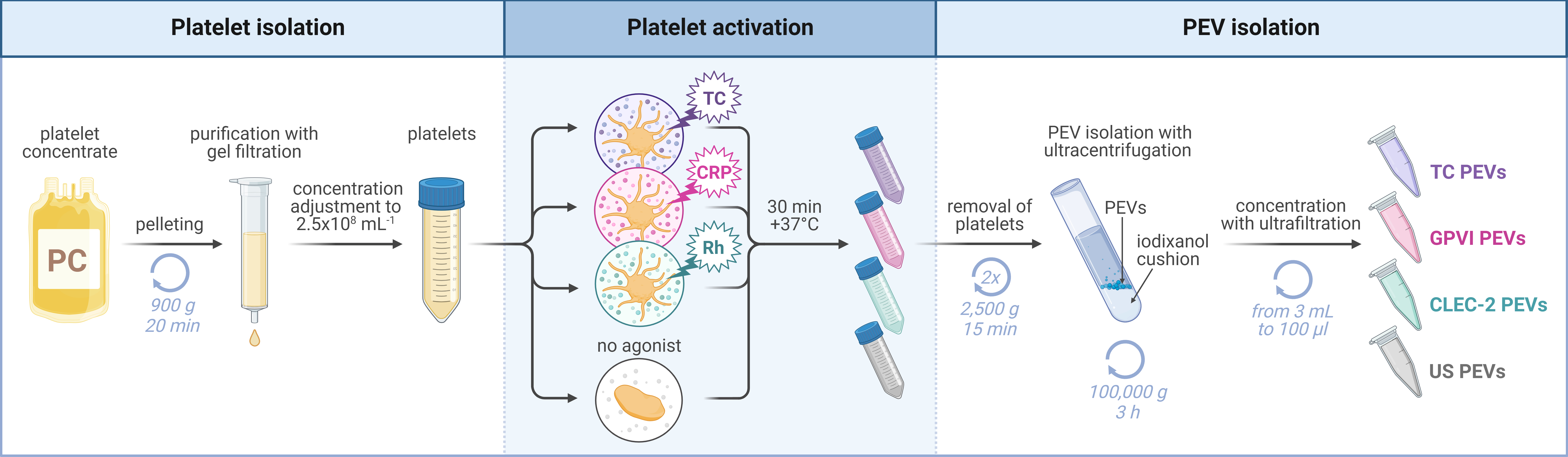

Supplement: Supplementary file 1 — Supporting Information [file JEV2-13-e12513-s001.zip › jev212513-sup-0002-FigureS1.jpeg]

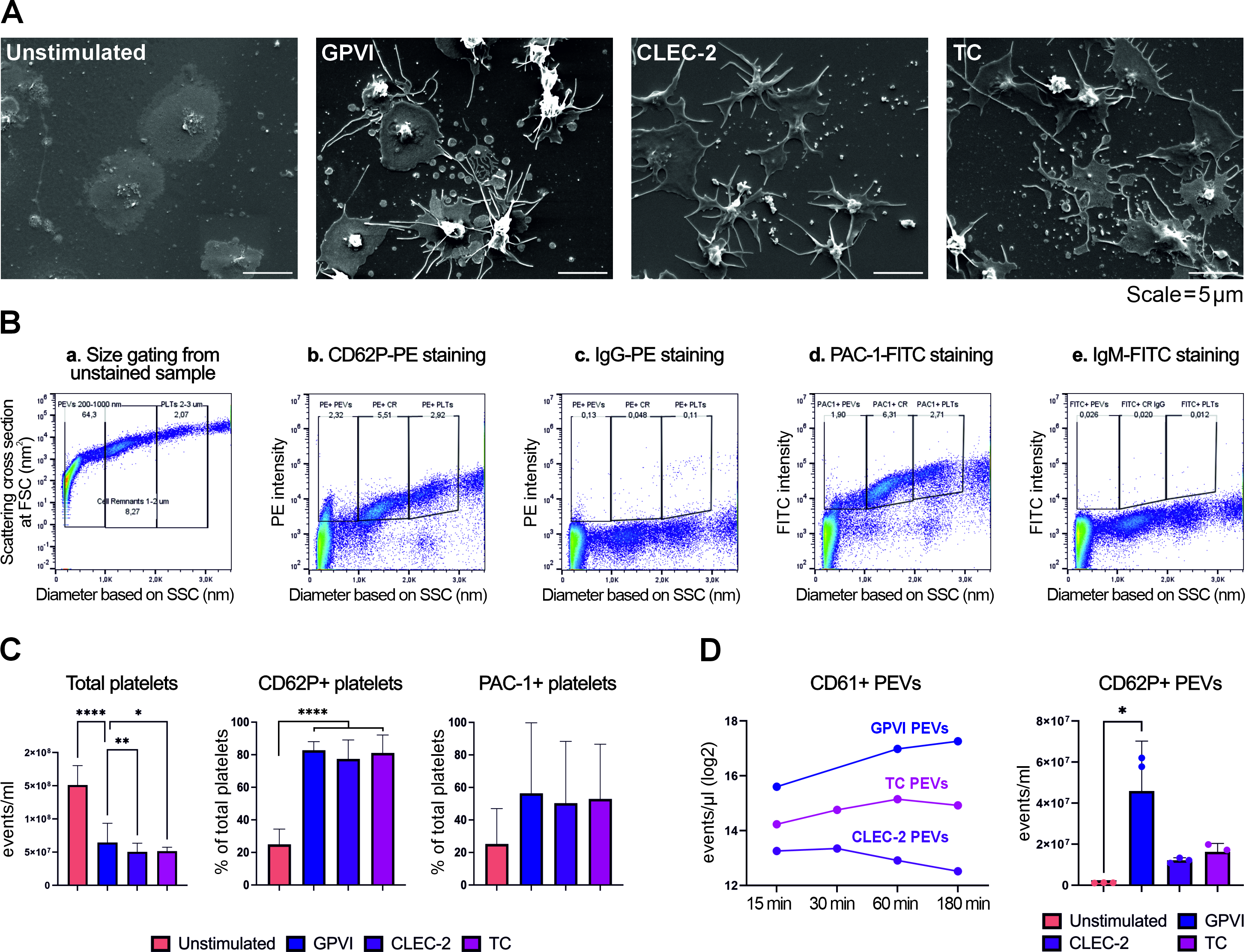

Supplement: Supplementary file 1 — Supporting Information [file JEV2-13-e12513-s001.zip › jev212513-sup-0003-FigureS2.tif]

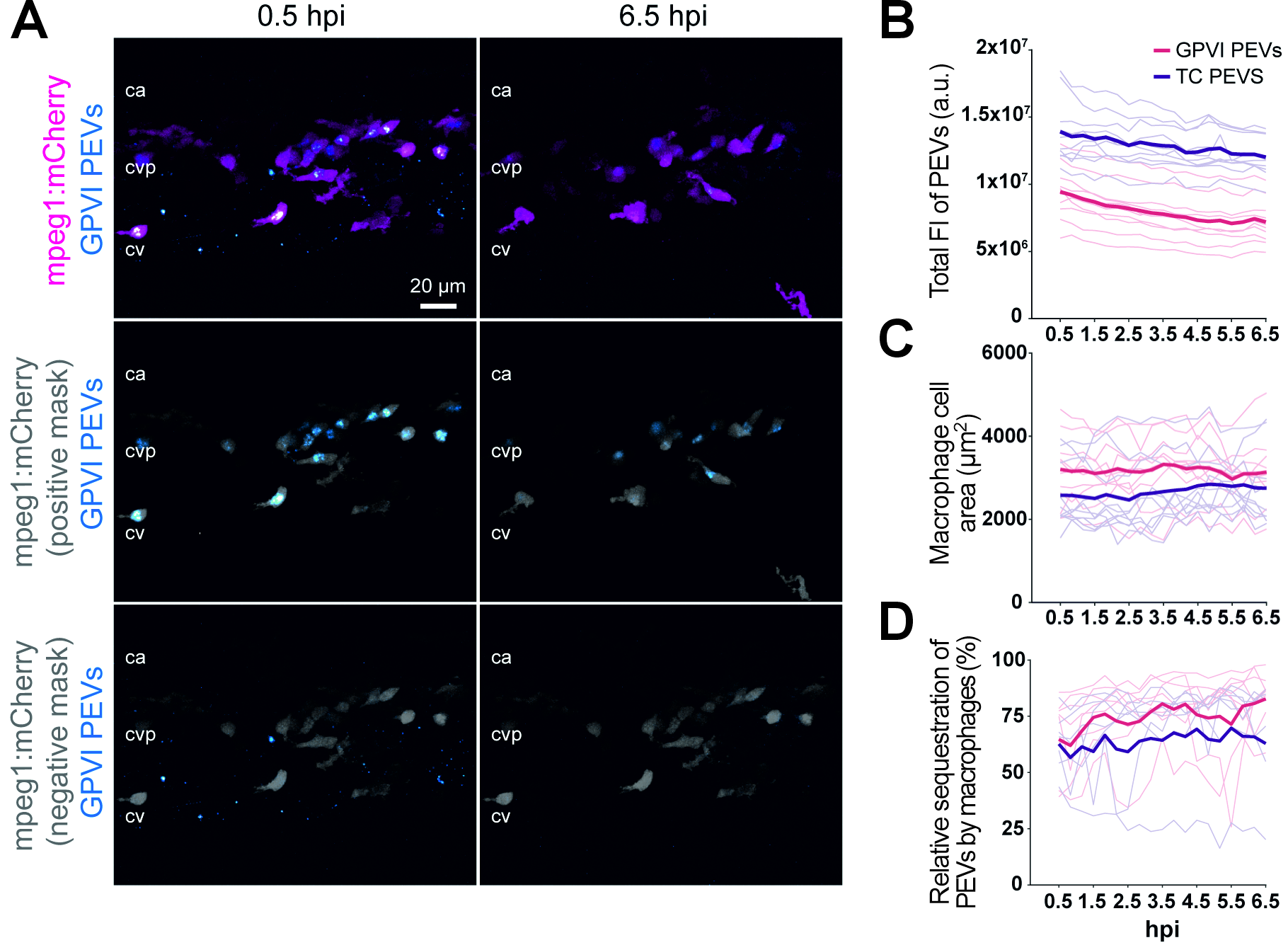

Supplement: Supplementary file 1 — Supporting Information [file JEV2-13-e12513-s001.zip › jev212513-sup-0004-FigureS3.tif]

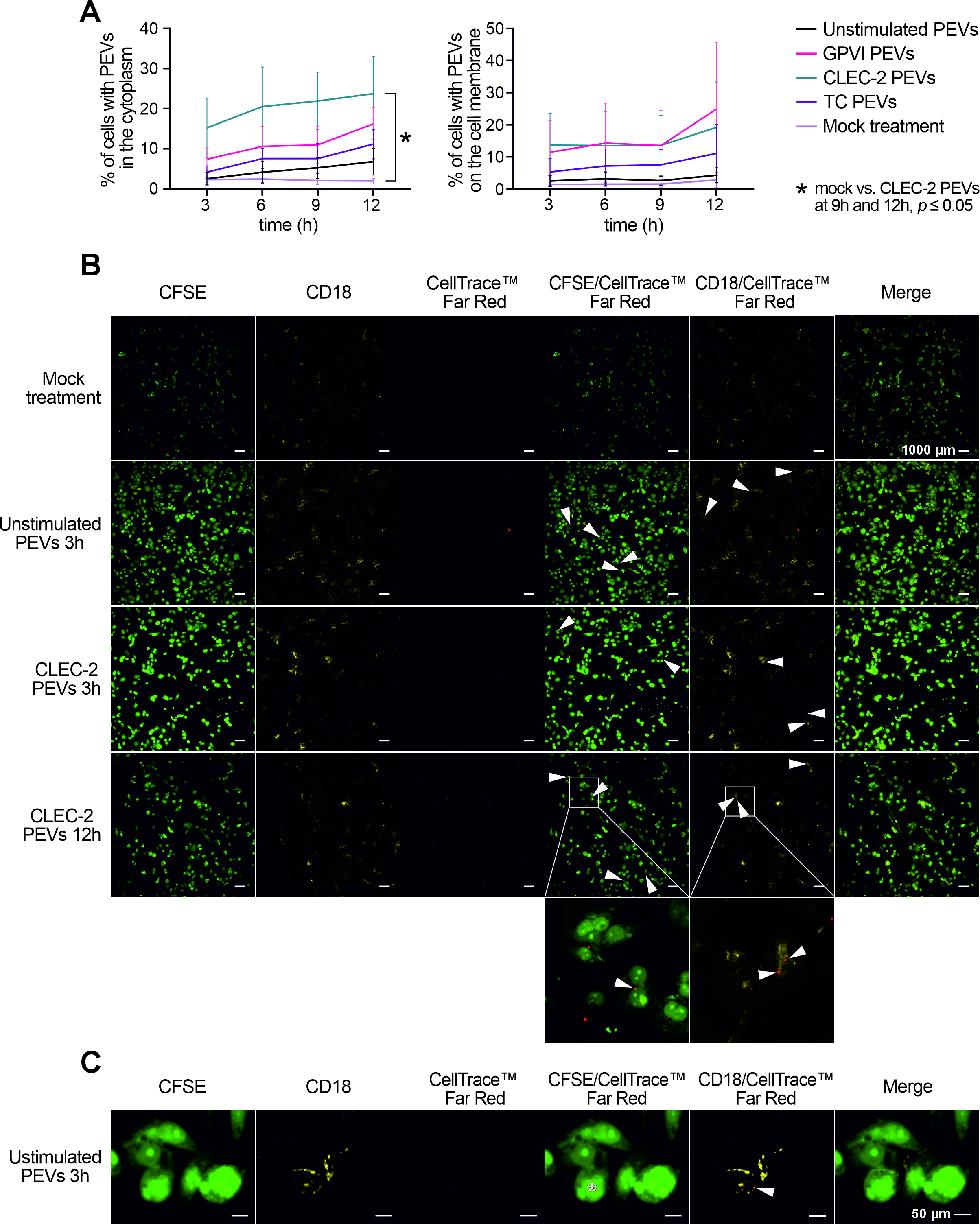

Supplement: Supplementary file 1 — Supporting Information [file JEV2-13-e12513-s001.zip › jev212513-sup-0005-FigureS4.tif]

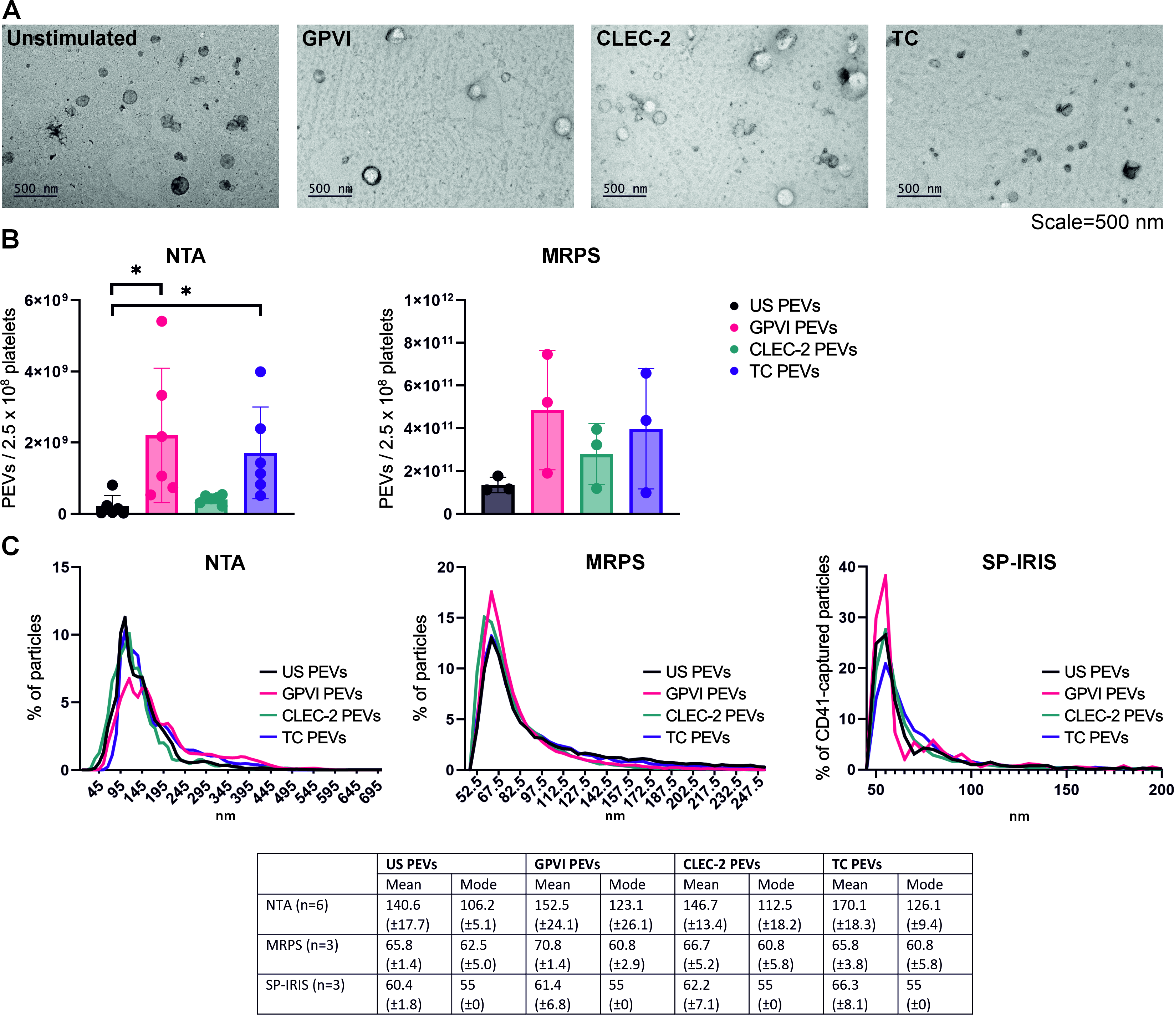

Supplement: Supplementary file 1 — Supporting Information [file JEV2-13-e12513-s001.zip › jev212513-sup-0006-FigureS5.tif]

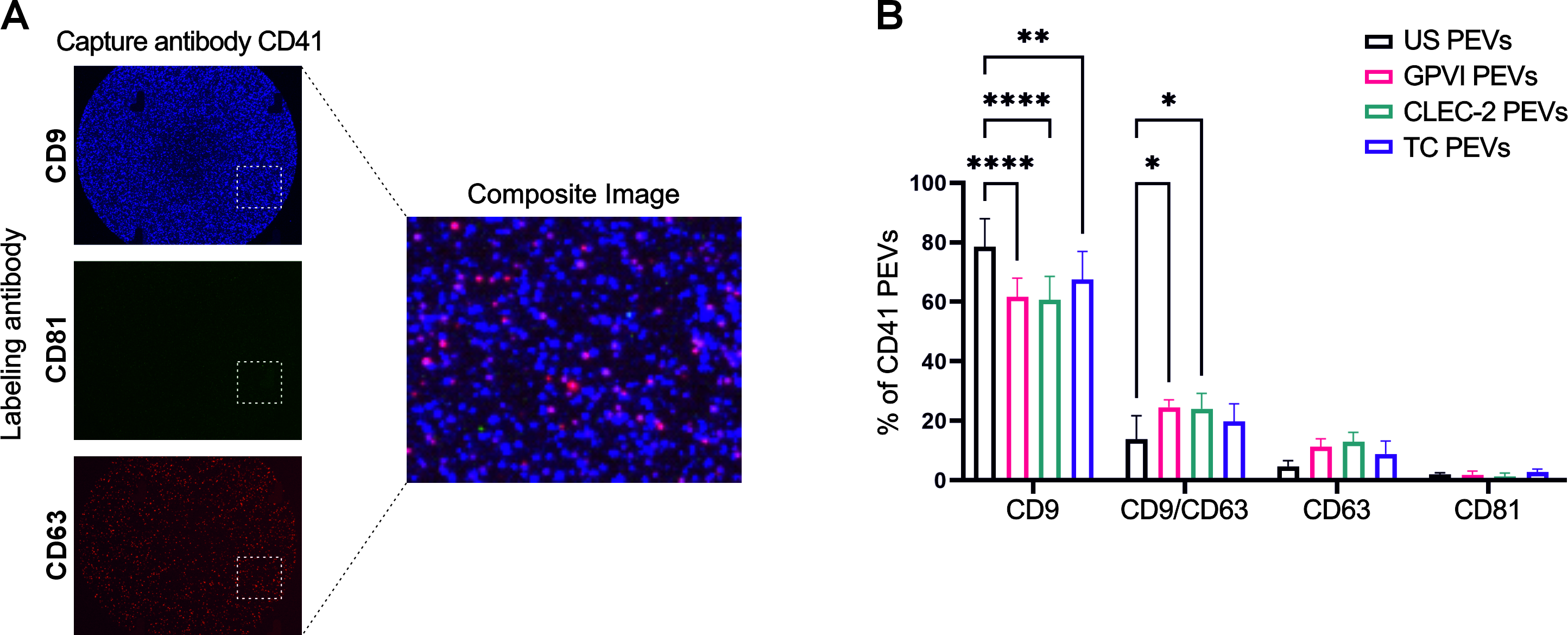

Supplement: Supplementary file 1 — Supporting Information [file JEV2-13-e12513-s001.zip › jev212513-sup-0007-FigureS6.tif]

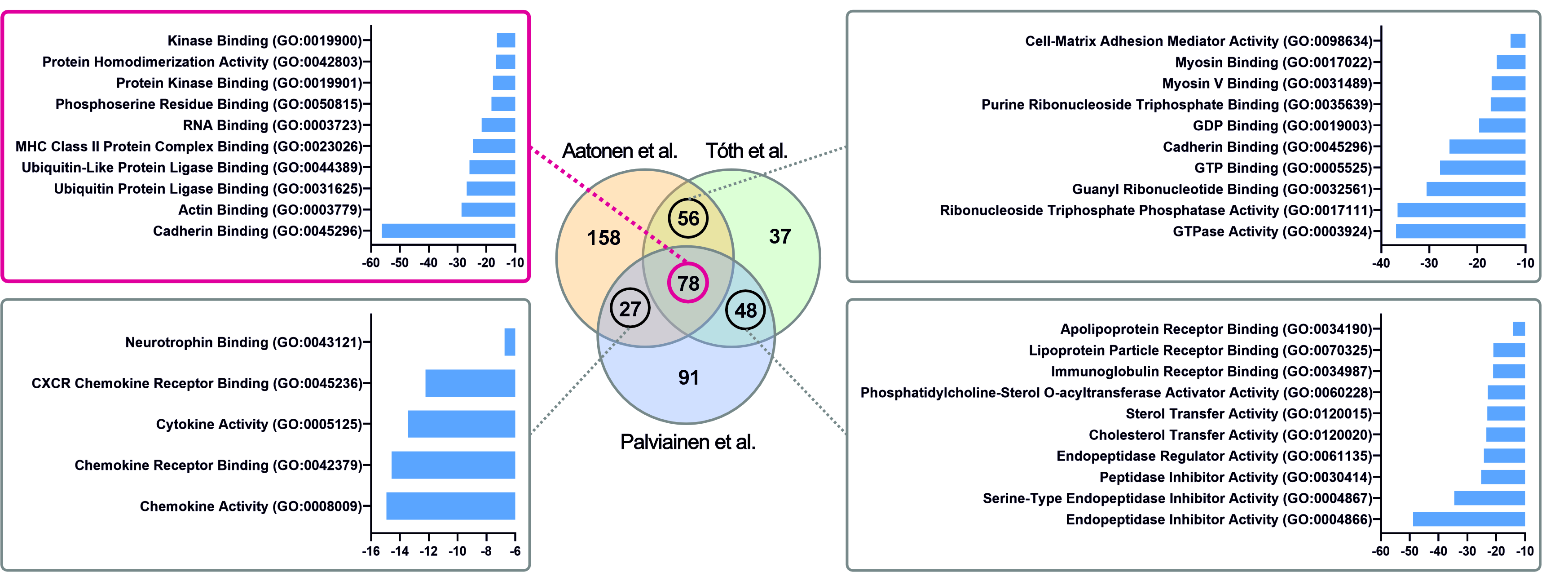

Supplement: Supplementary file 1 — Supporting Information [file JEV2-13-e12513-s001.zip › jev212513-sup-0008-FigureS7.tif]

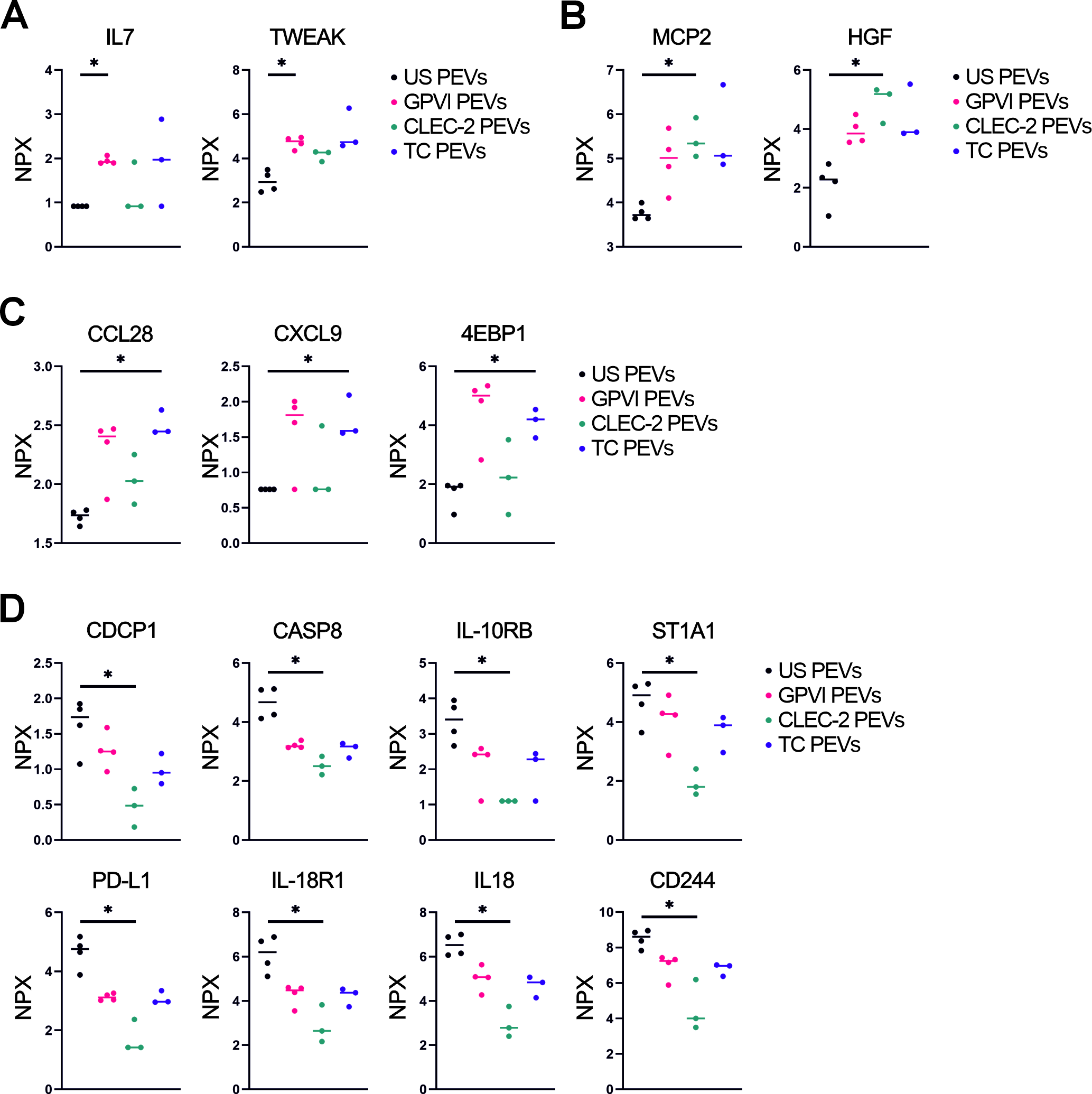

Supplement: Supplementary file 1 — Supporting Information [file JEV2-13-e12513-s001.zip › jev212513-sup-0009-FigureS8.tif]
